# Deciphering the Immune Complexity in Esophageal Adenocarcinoma and Pre-Cancerous Lesions With Sequential Multiplex Immunohistochemistry and Sparse Subspace Clustering Approach

**DOI:** 10.3389/fimmu.2022.874255

**Published:** 2022-05-19

**Authors:** Srinand Sundaram, Eun Na Kim, Georgina M. Jones, Shamilene Sivagnanam, Monika Tripathi, Ahmad Miremadi, Massimiliano Di Pietro, Lisa M. Coussens, Rebecca C. Fitzgerald, Young Hwan Chang, Lizhe Zhuang

**Affiliations:** ^1^ Medical Research Council (MRC) Cancer Unit, Hutchison-Medical Research Council (MRC) Research Centre, University of Cambridge, Cambridge, United Kingdom; ^2^ Department of Biomedical Engineering, Oregon Health and Science University, Portland, OR, United States; ^3^ Department of Cell, Developmental & Cancer Biology, Oregon Health and Science University, Portland, OR, United States; ^4^ Knight Cancer Institute, Oregon Health and Science University, Portland, OR, United States

**Keywords:** multiplex imaging, sparse subspace clustering, immune complexity, esophageal adenocarcinoma, Barrett’s esophagus

## Abstract

Esophageal adenocarcinoma (EAC) develops from a chronic inflammatory environment across four stages: intestinal metaplasia, known as Barrett’s esophagus, low- and high-grade dysplasia, and adenocarcinoma. Although the genomic characteristics of this progression have been well defined *via* large-scale DNA sequencing, the dynamics of various immune cell subsets and their spatial interactions in their tumor microenvironment remain unclear. Here, we applied a sequential multiplex immunohistochemistry (mIHC) platform with computational image analysis pipelines that allow for the detection of 10 biomarkers in one formalin-fixed paraffin-embedded (FFPE) tissue section. Using this platform and quantitative image analytics, we studied changes in the immune landscape during disease progression based on 40 normal and diseased areas from endoscopic mucosal resection specimens of chemotherapy treatment- naïve patients, including normal esophagus, metaplasia, low- and high-grade dysplasia, and adenocarcinoma. The results revealed a steady increase of FOXP3^+^ T regulatory cells and a CD163^+^ myelomonocytic cell subset. In parallel to the manual gating strategy applied for cell phenotyping, we also adopted a sparse subspace clustering (SSC) algorithm allowing the automated cell phenotyping of mIHC-based single-cell data. The algorithm successfully identified comparable cell types, along with significantly enriched FOXP3 T regulatory cells and CD163^+^ myelomonocytic cells as found in manual gating. In addition, SCC identified a new CSF1R^+^CD1C^+^ myeloid lineage, which not only was previously unknown in this disease but also increases with advancing disease stages. This study revealed immune dynamics in EAC progression and highlighted the potential application of a new multiplex imaging platform, combined with computational image analysis on routine clinical FFPE sections, to investigate complex immune populations in tumor ecosystems.

## Introduction

Esophageal cancer is the sixth most common cause of cancer death worldwide; it has two subtypes: esophageal squamous cell carcinoma and esophageal adenocarcinoma (EAC) (1). The incidence of EAC has increased almost 6-fold in western countries in the past decades where patients are typically diagnosed at an advanced stage; thus, the overall five-year survival rate is 15% (2).

EAC progression follows well-defined histopathological stages, metaplasia, low- and high-grade dysplasia (Dys), and eventually adenocarcinoma. The first precancerous metaplastic stage is termed Barrett’s esophagus (BE), where the squamous epithelium in the esophagus lining is replaced by an intestinal-like columnar epithelium. BE is not uncommon and is thought to affect one out of 100 people; it increases the risk of EAC by 30- to 60-fold and already bears a mutational load that is higher than some cancer types (3). Although the annual progression rate of BE to EAC is relatively low, BE could acquire more mutations and progress to Dys, which has a hallmark of p53 mutation and further increases the risk of EAC (4). It is noteworthy that approximately 80% of EAC patients are diagnosed *de novo*, with little chance to intervene (5, 6), highlighting the importance of the early detection strategy to tackle EAC. However, the challenge remains to accurately identify the biomarkers or signals that are present in early cancer or critical steps of cancer progression (7). The strongest known risk factor for BE development is gastroesophageal reflux disease (GERD), which leads to long-term inflammation in the gastroesophageal mucosa (8).

In general, chronic inflammation is associated with malignant progression in gastrointestinal tissues (9, 10). However, the precise molecular and cellular mechanisms at the gastroesophageal junction and the relationship to the development of BE and progression to EAC might be far more complicated. For example, esophagitis and BE, both benign conditions at the esophagus, have distinct inflammatory profiles in patients with reflux symptoms, where BE is dominated by a Th2-type response with high levels of IL-4 and IL-10, with elevated T-cell infiltration as compared with esophagitis and normal esophagus (11, 12). Lind and colleagues also reported that FOXP3 and RALDH1 are highly expressed in BE and suggested that BE, not esophagitis, is associated with dendritic cell populations and the retinoic acid pathway (13). In addition, PD-L1 and PD-L2 ligands that mediate T-cell effector responses are also observed in approximately 50% of BE and EAC cases based on immunostaining (14). In animal models, the overexpression of IL-1B, IL-6, and IL-8 promotes the development of BE-like phenotypes and malignant progression; the homozygous loss of IL-6 abolishes the metaplasia- and Dys-like phenotypes in IL-1B-overexpressing mouse models (15, 16). Interestingly, an increasing density of myeloid cells was also observed in the BE-like tissue of IL-1B/IL-8- overexpressing mice fed with a high-fat diet, correlated with accelerated malignant progression as compared with syngeneic mice on a control diet (16). Dendritic cells expressing CD1A or CD1C have also been reported in association with BE development toward malignancy, although their roles remain unclear (13, 17, 18).

It is noteworthy that a majority of published studies describing immune cells in BE and EAC utilized single-lineage biomarkers for previously identified populations, largely based on routine immunohistochemistry (IHC) methodologies and limited by specimen availability. Animal models of disease progression in general offer a wider availability of tissue and allow for the application of more complex technologies. However, a recent study with multi-omics single cell sequencing indicated that clinical BE in humans might not be accurately represented by mouse BE-like phenotypes (19). Based on these limitations and differences, we sought to directly examine clinical samples with a new methodology, allowing for a quantitative single-cell resolution to identify immune cell populations in patient samples.

In this study, we combined multiplex tissue imaging and a computational image analysis pipeline to study immune complexity in EAC and its precancerous lesions. We assembled a cohort of endoscopic mucosal resection (EMR) specimens from 6 patients and analyzed 40 regions of interest (ROIs) reflecting normal esophagus, BE, Dys, and EAC using sequential mIHC that allows for detection of 10 biomarkers on a single FFPE slide (20). We implemented an automated staining protocol and implemented an image analysis pipeline including image co-registration, nuclear segmentation, and marker quantification to extract single cell-based data (21). Resultant data were then analyzed by two methods, including the manual hierarchical gating of image cytometry profiles and automated cell phenotyping based on sparse subspace clustering-based approaches (22), to characterize changes in the immune landscape and the spatial distribution of cells during the progression of BE to Dys and EAC and to explore biomarkers that could facilitate early detection of EAC and/or stratify the risks of disease progression.

## Methods and Materials

### Sample Collection and Annotation

EMR samples were collected during clinically indicated endoscopic procedures in patients referred to Cambridge University Hospitals NHS Trust (Addenbrooke’s Hospital, Cambridge, United Kingdom) for the treatment of BE-related neoplasia. The study was approved by the Institutional Ethics Committees, and all subjects gave individual informed consent for the use of their tissue samples for research purposes (REC 01/149). Briefly, after the endoscopic procedure, EMR specimens were pinned down on cardboard using 20 mm Agani needles and fixed overnight in formalin and processed for histology as per clinical standard. Approximately 5 μm thick FFPE sections were cut, and adjacent sections were stained for hematoxylin and eosin (H&E) for histopathological evaluation.

The H&E slides were scanned and printed out. Two experienced specialist gastrointestinal pathologists, MT and AM, independently assessed the H&E slides using the definitions and histological criteria for normal esophagus, Barrett’s metaplasia, the grades of Dys, and esophageal adenocarcinoma recommended by The Royal College of Pathologists (23) and guidelines by the British Society of Gastroenterology on the diagnosis and management of Barrett’s esophagus (24). There were no discrepancies between the two pathologists when reviewing the samples presented in this study. Indefinite Dys was excluded; high and low grades of Dyswere grouped together as Dys. All local pathological grades were marked on the corresponding H&E printout. ROIs were then determined based on the marked H&E printout. Unstained FFPE slides that were adjacent to the assessed H&E slides were selected to proceed for mIHC.

### Automated mIHC Staining

The staining was carried out on Bond RX platform (Leica System), following modified standard ‘IHC protocol F’ and Heat Induced Epitope Retrieval 1 (HIER1) for 20 min using the BOND Polymer Refine Detection kit. The ‘IHC protocol F’ was modified by 1) inserting three extra 5 min washing steps after HIER; 2) replacing the step ‘Mixed DAB Refine’ with ‘Bond Open Container’, which was supplied with freshly made 3-Amino-9-Ethylcarbazole (AEC); and 3) removing the step of ‘Hematoxylin’. One cycle of mIHC was carried out as one staining protocol, following the standard operation of the manufacturer’s protocol, except a) in the first cycle, the ‘Dewax’ step was selected, and b) in the last cycle, the ‘Hematoxylin’ step was added back as a sole staining step.

After each cycle of staining, slides were unloaded from the Bond machine and temporarily mounted in Tris-Buffered Saline with 0.1% Triton X-100 (TBST) and imaged using Zeiss AxioScan Z1 at 20x brightfield. After imaging, AEC was removed by ethanol by 2 brief washes in distilled water, 1 wash in 70% ethanol, and 1 wash in 100% ethanol for 3.5 min. The slides were then rehydrated through 2 min incubation in 70% ethanol, 1 min incubation in 30% ethanol, and then 4 washes in distilled water. The slides were then left to rest in TBST for the next cycle.

### Raw Image Process and Manual Gating

**Figure 1:**
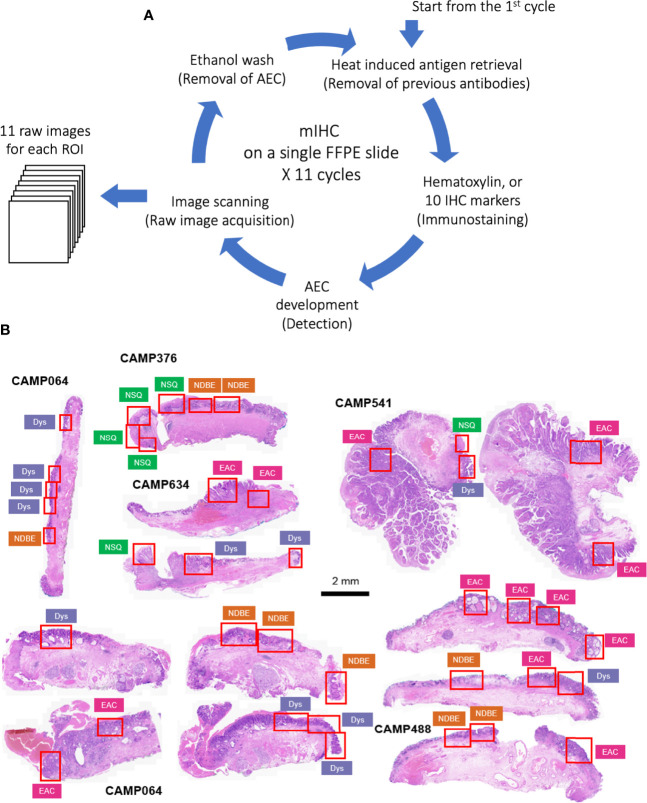
**(A)** Schematic of staining cycles of mIHC; **(B)** H&E image of EMR tissue cohort and ROIs of different disease grades. NSQ, normal squamous esophagus; NDBE, non-dysplastic Barrett’s esophagus; Dys, dysplasia; EAC, esophageal adenocarcinoma.

The raw images were generated in Carl Zeiss Image (CZI) format with resolution of 0.22 µm per pixel by Zeiss AxioScan Z1 at 20x brightfield. The raw CZI images were processed using the Zeiss software, Zen Lite, to generate ROI images in TIFF format according to the pathologist’s grading (**Figure 1B**). Each ROI has 11 raw Tag Image File Format (TIFF) images including nuclear staining and 10 biomarkers.

**Figure 2:**
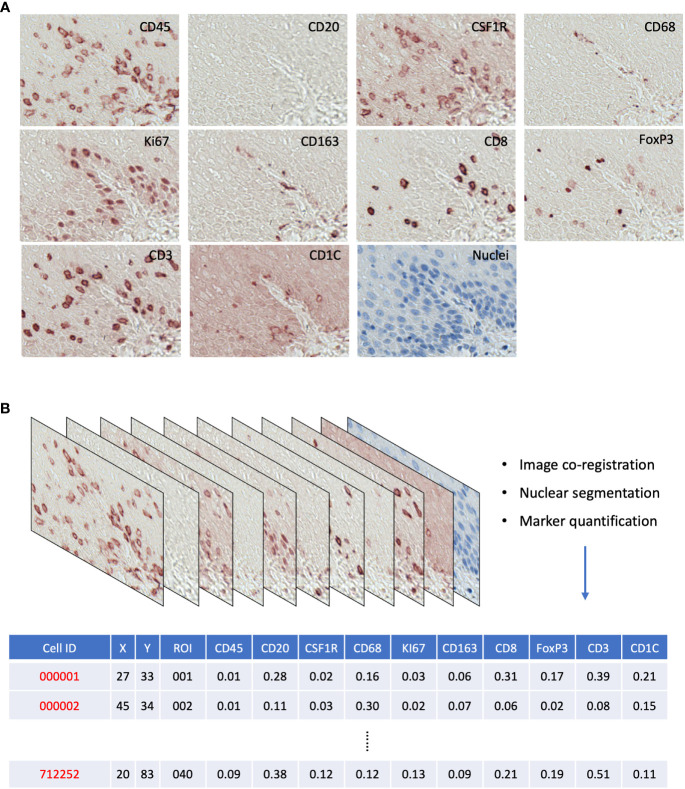
**(A)** Raw AEC staining for each marker and hematoxylin staining for nuclei. **(B)** Schematics of raw image processing and raw data format, each row is a cell with its ID and ROI; *x*,*y* coordinates; and quantitative expression values for each mIHC marker.

For each ROI, image coregistration was performed using a MATLAB-based script: ‘register_crop_batch4tif_CRUK_OHSU_mIHC.m’, which generated coregistered images shown in **Figure S2B**. The deconvolution of the 10 marker images (AEC stained) and the nuclear segmentation of the nuclear image were performed *via* the ImageJ macro: ‘AEC_CMYK_Seg_Overlay_Batch_CRUK_OHSU_mIHC.ijm’. This pipeline generated a simulated IHC image of each marker (**Figure S3A**) and monochrome images for simulated immunofluorescence (**Figure S3B**). It also generated the nuclear mask, a TIFF image with each single cell segmented, and a raw CellProfiler Output (CPOUT) file for FCS Express™ Image Cytometry (*De Novo* Software, Los Angeles, CA, USA) for manual gating, which were described in (15) and in **Figure S4**. The cell numbers of all gates were exported as comma-separated values (CSV) files (**Figure 2B**) for the analysis of cell density and proportion and then used in the sparse subspace clustering (SSC) approach and spatial analyses.

### Automated Cell Phenotyping Using Sparse Subspace Clustering Algorithm

In this approach, to identify the groups of cells with similar features, we facilitated the characterization of biologically significant cellular characteristics where representative data points are selected as landmarks, representing the original data points as linear combinations; *X*=[ *x*_1_, ⋯, *x*_*N*_ ] is an *m* × *N* data matrix where *x_i_* represents a feature vector corresponding to the i*-th* cell, *m* represents the dimensionality of a feature vector *x_i_*, and *N* is the number of segmented cells.

The main idea of SSC takes advantage of the self-expressiveness property of the data; for instance, each data point in a union of subspaces can be efficiently reconstructed by a combination of other points in the dataset. More precisely, each data point (or the expression of proteins for a segmented cell) *x_i_* can be written as *x_i_* = *X c_i_* where *c*_*i*_≜[ *c*_*i*1_ *c*_*i*2_⋯*c*_*N*_ ]^*T*^ and the constraint *c_ii_* = 0 eliminates the trivial solution of writing a point as a linear combination of itself. In other words, the matrix of data points (i.e., the expression of proteins of all segmented cells) *X* is a self-expressive dictionary in which the expression of the proteins of an individual cell can be written as a linear combination of the expression of proteins of other cells. In general, as the representation of *x_i_* in the dictionary *X* is not unique, among all solutions, we seek a sparse solution, *c_i_*, whose nonzero entries correspond to the data points from the same subspace as *x_i_*. By doing this, we identify a subspace-sparse representation where a sparse representation of a data point finds points from the same subspace where the number of the nonzero elements corresponds to the dimension of the underlying subspace. One can restrict the set of solutions by minimizing an objective function such as the *l*_1_-norm of the solution as follows:


$$\text{min}{∥c_{i}∥}_{q}subject\;tox_{i}=X\;c_{i},c_{ii}=0$$

After solving the proposed optimization program, we obtain a sparse representation for each data point whose nonzero elements ideally correspond to points from the same subspace. The next step of the algorithm is to infer the clustered group of the data into different subspaces using the sparse coefficients. The clustering of data into subspaces then follows by applying spectral clustering to the similarity graph in which the nodes that correspond to points from the same subspace are connected to each other and there are no edges between the nodes that correspond to points in different subspaces (k). SSC manages complexity by selecting a few representative points as landmarks so that the spectral embedding of the data can be efficiently computed with the landmark-based representation.

### Spatial Pattern and Neighborhood Enrichment Analyses

We used the Single-Cell Image Analysis Package (SCIMAP) open-source python library (https://github.com/labsyspharm/scimap) to quantify the average shortest distance between reference and target cells (scimap.tl.spatial_distance) and implemented neighborhood enrichment analysis to compute how likely cell types are found next to each other compared to a random background (scimap.tl.spatial_interaction). This uses a permutation test to compare the number of interactions between all cell types in a given image to that of a matched control containing randomized cell phenotypes. Thus, it enables the unbiased and systematic study of cell–cell interactions present in all the tissues of a sample cohort. By doing this, it determines the significance of cell–cell interactions, reveals enrichments (red) or depletions in cell–cell interactions (blue) that are indicative of cellular organization, and statistically non-significant results (gray). For parameters, we used the k-nearest neighbor algorithm (knn = 10) to identify the neighbors for every cell and the number of permutations with 1,000 as default. P-values are calculated by subtracting the permuted mean from the observed mean divided by the number of permutations as described in (25). For analyzing multiple images together, a cluster map shows the average cell–cell interaction across all images.

### Statistics

For **Figures 4**, **5**, **7**, we used the Mann–Whitney–Wilcoxon test two-sided with Bonferroni correction. In a given cell population, we treated its density in the four different stages: normal esophagus with squamous epithelium (NSQ), non-dysplastic BE (NDBE), Dys, and EAC, as four independent groups. One sample point represented one ROI, and the p-value was calculated individually between two selected stages, e.g., NSQ vs. NDBE or Dys vs. EAC, whereby each stage contained a number of non-overlapping ROIs. For **Figures S6–S8**, those cell subsets have high variations, whereby we did not particularly aim to investigate the change between each disease stage. We therefore chose the nonparametric Kruskal–Wallis one-way ANOVA test in R to determine if a given cell subset is statistically significantly different by at least one disease stage. For **Figures 8** **–F**, we applied the Mann–Whitney-Wilcoxon test two-sided with Bonferroni correction for multiple comparison with the 5% significance level [open-source python library (26)].

## Results

### Establishment of Automated Immunohistochemistry Staining and Computational Image Analysis Pipelines

The principles underlying mIHC have been previously described (22, 27). Briefly, each signal marker is detected by chromogenic-based (3-amino-9-ethylcarbazole, AEC) IHC in a sequential and multi-cycle manner, followed by digital imaging. After imaging, AEC is removed by ethanol and antibodies are stripped by heated citrate buffer before entering the next cycle (**Figure 1A**). Here, we modified the process to enable automated staining using the Leica BOND Automated IHC Stainer platform where each cycle could be performed as one standard staining program, and up to 30 slides could be stained in the same batch. We also confirmed complete removal of markers from previous cycles using the BOND standard heat-induced epitope retrieval step (**Figure S1**, **Table 1** and Method).

**Table 1:** Antibodies used in mIHC.

Target	Supplier	Product Reference	Host species	Isotype	Dilution factor
CD45	Cell Signalling	13917	Rabbit	IgG	200
CD20	Abcam	ab9475	Mouse	IgG2a	100
CSF1R	Abcam	ab183316	Rabbit	IgG	400
CD68	Abcam	ab783	Mouse	IgG3	50
KI67	Abcam	ab16667	Rabbit	IgG	200
CD3	Abcam	ab16669	Rabbit	IgG	150
CD8	Dako	M7103	Mouse	IgG1	150
CD163	Thermofisher	MA5-11458	Mouse	IgG1	100
FOXP3	Abcam	ab20034	Mouse	IgG1	100
CD1C	Abcam	ab156708	Mouse	IgG1	150

We generated a series of digitized images for each marker representing all the EMR tissues, which were assessed independently by two pathologists for local disease grades. This allowed us to identify and acquire 40 ROIs spanning from an NSQ, NDBE, Dys, and EAC (**Figure 1B**; **Table 2**). Each ROI measured approximately from 0.5 × 1 mm to 1 × 2 mm, contained 11 single channel images that corresponded to the nuclear staining, and 10 biomarkers including CD45, CD3, CD8, FOXP3, CD20, CD68, CSF1R, CD163, CD1C, and KI67, respectively (**Figure 2A**). Previously, images were co-registered manually by identifying a “fiducial” point through all images. Here, we established an automated image registration algorithm based on the distinct tissue pixel gradients of the ROI and feature matching to coregister the images of different markers of the same ROI (21, 28). In the computational process, first, we extracted feature descriptors, matched features by using their descriptors, and retrieved the locations of corresponding points for each image. Then, we estimated transformation corresponding to the matching point pairs and recovered the scale and angle by using the geometric transformation. This algorithm enabled a fully automated coregistration and batch processing (**Figure S2**). To assess the 10 biomarkers and their expression on every single cell, coregistered image stacks were processed using a watershed-based segmentation in FIJI (Fiji is Just ImageJ), which segmented single cells based on the hematoxylin nuclear staining, and then quantified the chromogenic intensities of AEC staining for each biomarker. Image analysis pipelines also generated multi-channel-merged IHC images that were reassessed by pathologists (**Figure S3A**), with monochrome images used to generate multi-channel images to visualize selected biomarkers (**Figure S3B**). Using the automated IHC staining and automated computational image analysis pipelines, we acquired raw data for 712,252 cells from the 40 ROIs with quantitative expression values from 10 biomarkers on a single-cell basis, including the cells’ shape–size measurement and spatial coordinates within the tissue (**Figure 2B**).

**Table 2:** EMR sample cohort and ROI annotation.

Patients	Pathological grades present in tissues	Number of ROIs
CAMP067	NDBE, Dys, EAC	n = 9 (NDBE: 3; Dys: 4; EAC: 2)
CAMP488	NDBE, Dys, EAC	n = 10 (NDBE: 3; Dys: 1; EAC: 6)
CAMP541	NSQ, Dys, EAC	n = 5 (NSQ: 1; Dys: 1; EAC: 3)
CAMP064	NDBE, Dys	n = 5 (NDBE: 1; Dys: 4)
CAMP376	NSQ, NDBE	n = 6 (NSQ: 4; NDBE: 2)
CAMP634	NSQ, Dys, EAC	n = 5 (NSQ: 1; Dys: 2; EAC: 2)

All EAC samples here are at the stage of T1a.

### Immuno-Phenotyping of mIHC Data Revealed Changes of T Regulatory Cells, CD163^+^ Myelomonocytic Cells, and CD8^+^ T Cells in the Progression of BE to EAC

To identify key immune cell populations from the ROIs, we loaded raw data into FCS Express™ Image Cytometry (see *Materials and Methods*). Similar to flow cytometry, each data point corresponds to a cell projected in a biaxial plot that allows for quantitative assessment and selection (gating) based on the markers of choice. In addition, spatial gates could be directly annotated on the tissue images to select cells within a specific region. We therefore specifically focused on the BE/EAC infiltrating immune cells *via* gating cells within the BE or EAC epithelium and adjacent 200 µm wide stromal margin in all ROIs (**Figure S4**). We then gated and assessed 17 key cell populations of lymphoid and myeloid lineages by adapting the previously reported gating strategies (20, 27) (**Figure S4**; **Table 3**), including but not limited to CD45^+^ total immune cells, CD45^+^CD3^+^ T cells, CD45^+^CD3^+^CD8^-^FOXP3^+^ T regulatory (Treg) cells, CD45^+^CD3^-^CD20^+^ B cells, CD45^+^CD3^-^CD20^-^CD1C^-^CD68^+^CSF1R^+^ myelomonocytic subsets, and the reported myeloid dendritic cell populations (13, 17, 18), marked by CD45^+^CD3^-^CD20^-^CD1C^+^ (hereinafter referred to as the CD1C^+^ subset). Gated cells were then visually overlaid onto original IHC images to confirm gating accuracy (**Figure S5**).

**Table 3:** Marker combination used in manual gating in image cytometry.

CD45^+^	Pan immune cells
CD45^+^CD3^+^	T cells
CD45^+^CD3^+^CD8^+^	CD8^+^ T cells
CD45^+^CD3^+^CD8^+^KI67^+^	Proliferating CD8 T cells
CD45^+^CD3^+^CD8^-^	CD8^-^ T cells
CD45^+^CD3^+^CD8^-^FOXP3^+^	T regulatory cells
CD45^+^CD3^+^CD8^-^FOXP3^+^KI67^+^	Proliferating T reg cells
CD45^+^CD3^+^CD8^-^FOXP3^-^	Other Th cells
CD45^+^CD3^-^CD20^+^	B cells
CD45^+^CD3^-^CD20^-^	Presumptive myeloid lineages
CD45^+^CD3^-^CD20^-^CD1C^+^	CD1C^+^ myeloid dendritic cells
CD45^+^CD3^-^CD20^-^CD1C^-^CD68^+^CSF1R^+^	Myelomonocytic cells
CD45^+^CD3^-^CD20^-^CD1C^-^CD68^+^CSF1R^-^	Monocytes, macrophages, fibrocytes
CD45^+^CD3^-^CD20^-^CD1C^-^CD68^+^CSF1R^+^CD163^+^	CD163^+^ myelomonocytic cells
CD45^+^CD3^-^CD20^-^CD1C^-^CD68^+^CSF1R^+^CD163^-^	CD163^-^ myelomonocytic cells

**Figure 3:**
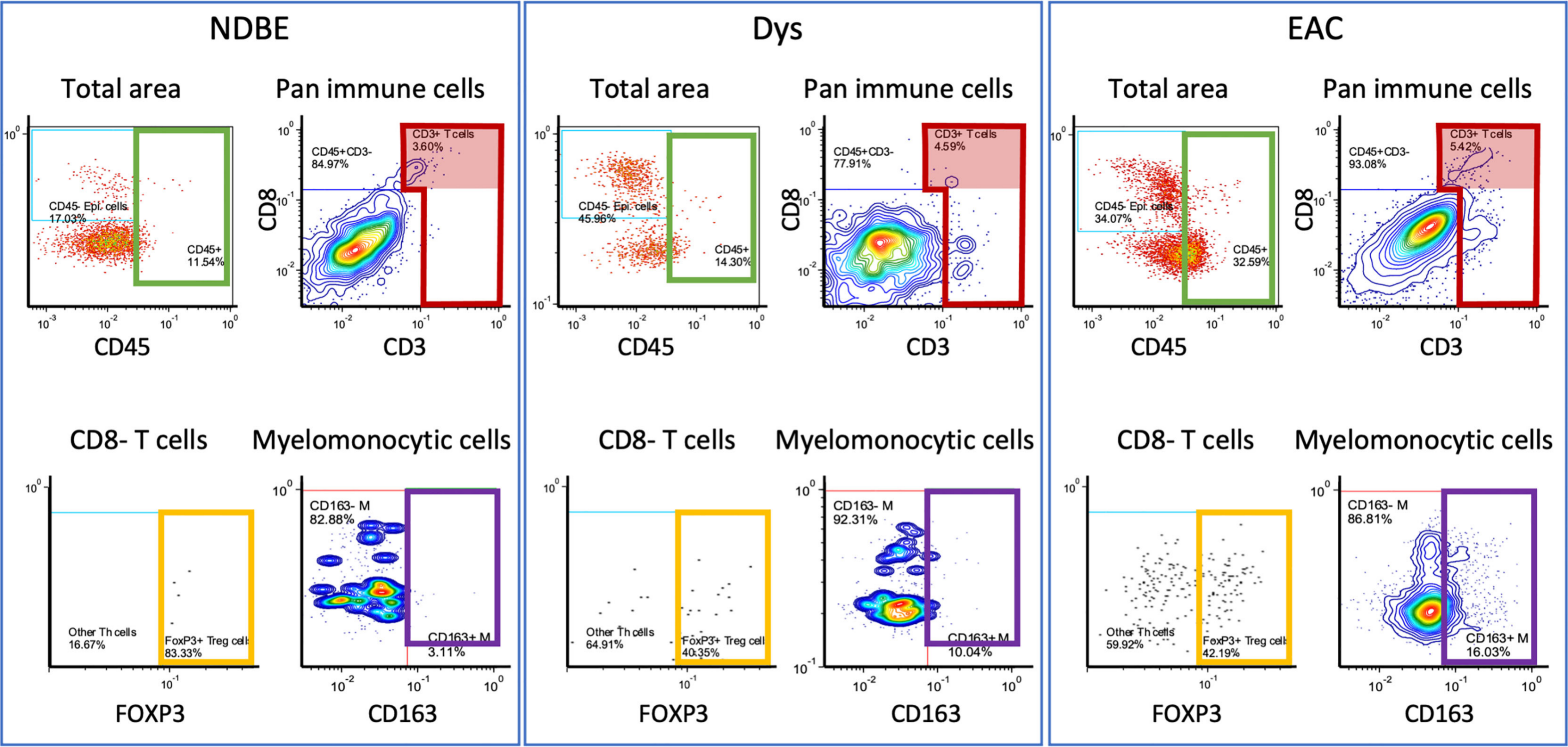
Representation of manual gating in the image cytometry of NDBE, Dys, and EAC: green gate: CD45 cells for total cells for selected area; red gate: CD3^+^ cells from total CD45^+^ immune cells; red highlighted areas: CD3^+^CD8^+^ cells; yellow gate: FOXP3^+^ Treg cells from CD45^+^CD3^+^CD8^-^ T cells; and purple gate: CD163^+^ myelomonocytic cells from total myelomonocytic cells (CD45^+^CD3^-^CD20^-^CD1C^-^CD68^+^CSF1R^+^). Note the change of gated cells in different disease stages, see **Figure S4** for gating strategy.

**Figure 4:**
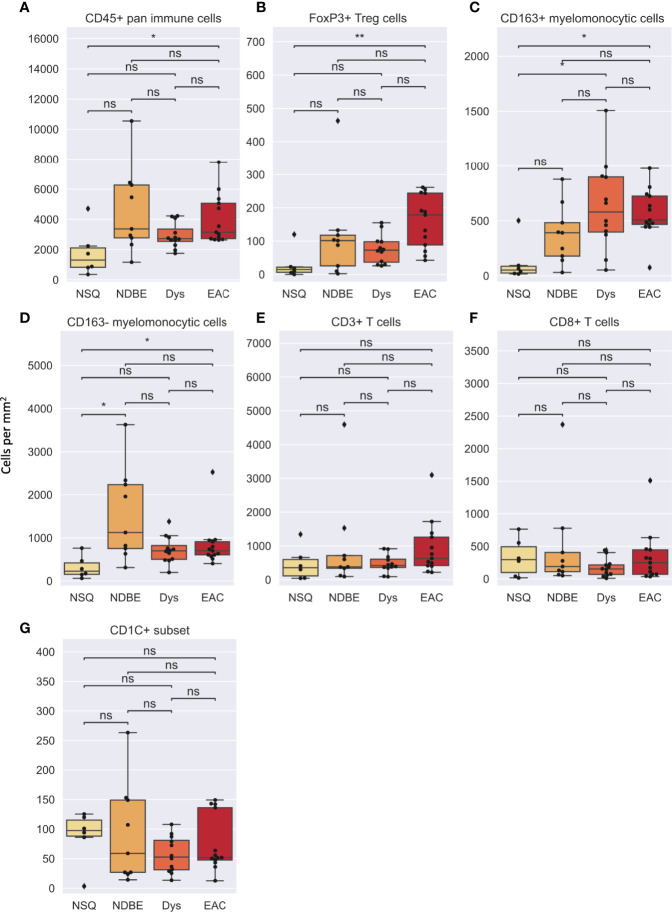
Cell density per mm^2^ of various gated cell subsets based on Image Cytometry at different disease stage. **(A)** CD45^+^ pan immune cells, **(B)** FoxP3^+^ Treg cells, **(C)** CD163^+^ myelomonocytic cells, **(D)** CD163^–^ myelomonocytic cells, **(E)** CD3^+^ T cells, **(F)** CD8^+^ T cells and **(G)** CD1C^+^ subset. Statistics: Mann-Whitney-Wilcoxon test two-sided with Bonferroni correction: ns (not significant): p > 0.05; *p ≤ 0.05; **p ≤ 0.01.

In image cytometry gating profiles, we observed that the total cells were shifting toward higher CD45^+^ density with disease progression; there was also an obviously increased presence of CD45^+^CD3^+^CD8^-^FOXP3^+^ Treg cells and the CD45^+^CD3^-^CD20^-^CD1C^-^CD68^+^CSF1R^+^CD163^+^ myelomonocytic subset (hereinafter referred to as CD163^+^ myelomonocytic cells) (**Figure 3**). We then plotted the cell densities (cell number per square millimeter) of all populations according to the disease grade of each ROI (**Figures 4** and **Figure S6**). Among all, the total CD45^+^ immune cells exhibited a clear increasing trend as the disease advanced (**Figure 4A**), which was in line with the overall pro-inflammatory microenvironment of BE/EAC development (29). Treg cell density showed a steady increase from NSQ to EAC (**Figure 4B**), where two significant increases were observed in NSQ (approx. 30 cells/mm^2^) versus NDBE (approx. 115 cells/mm^2^) and from Dys (approx. 76 cells/mm^2^) versus EAC (approx. 160 cells/mm^2^). A similar increasing trend was also observed in CD163^+^ myelomonocytic cell subsets, which reached the highest level in Dys (580 cells/mm^2^) and appeared to remain constant in EAC (**Figure 4C**). The overall trend in the CD CD163^–^ myelomonocytic cell subset was less clear, whereby it had significantly higher density in all disease grades compared with NSQ but appeared to reach the highest level in NDBE (**Figure 4D**). Interestingly, the abundance of both total T cells (CD45^+^CD3^+^, **Figure 4E**) and CD8^+^ T cells (CD45^+^CD3^+^CD8^+^, **Figure 4F**) did not show a clear trend, with similar observations made in the CD1C^+^ subset, too (**Figure 4G**).

**Figure 5:**
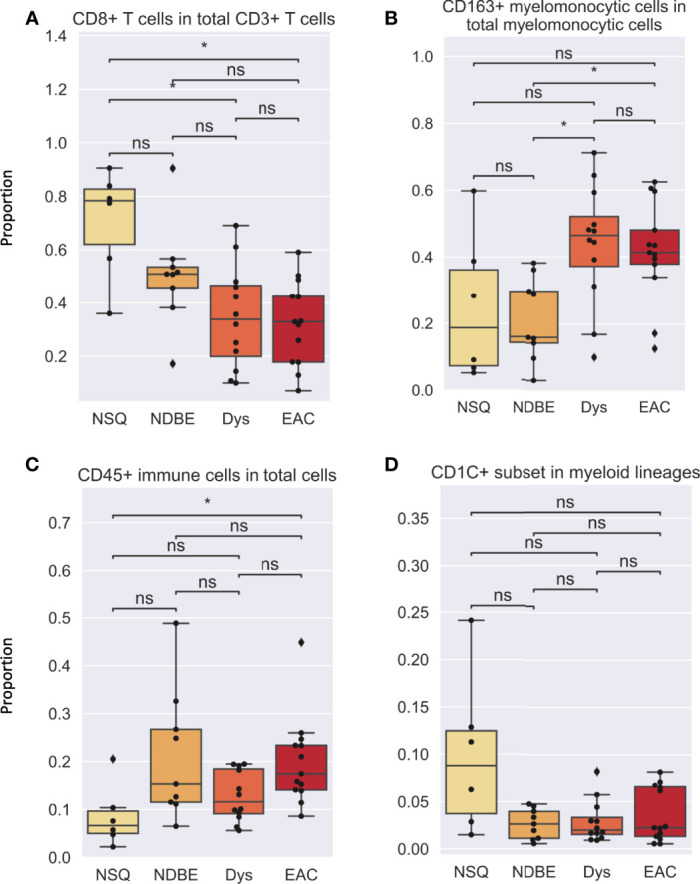
Cell proportion of various gated cell subsets based on Image Cytometry at different disease stage. **(A)** CD8^+^ T cells in total CD3^+^ T cells, **(B)** CD163^+^ myelomonocytic cells in total myelomonocytic cells, **(C)** CD45^+^ immune cells in total cells, **(D)** CD1C^+^ subset in myeloid lineages. Each datapoint represent one ROI. Statistics: Mann-Whitney-Wilcoxon test two-sided with Bonferroni correction: ns (not significant): p > 0.05; *p ≤ 0.05.

In addition to cell density, the immune complexity is also reflected by cell composition; we therefore assessed for each cell group (**Figures 5**,**S7**). Notably, the proportion of CD8^+^ T cells decreased dramatically as the disease advanced (**Figure 5A**). In addition, the proportion of CD163^+^ myelomonocytic cell subset in total myelomonocytic cells significantly increased, from approx. 15% in normal NSQ and NDBE to approx. 50% in Dys and EAC (**Figure 5B**). The proportion of total CD45^+^ immune cells (**Figure 5C**) and Treg cells (**Figure S7**) were in line with the cell densities that had a significantly increasing trend, but no clear trend was observed in the CD1C^+^ subset (**Figure 5D**).

### Automated Cell Phenotyping Using Sparse Subspace Clustering Approach

The hierarchical gating strategy in image cytometry was useful in identifying immune cell populations from our multiplex image dataset. However, it relied on *a priori* information, such as canonical or known marker combinations and expertise in gating and confirming the cell classifications for each ROI (**Figures S4, S5**). In addition, because the gating was performed manually on biaxial plots, the data were only assessed sequentially for up to two markers at a time; therefore, in high-dimensional data, such as this dataset, other important markers that describe cell phenotypes may be missed. In the case of using multiple biomarkers to evaluate more than 10 parameters, manual analysis *via* this gating strategy becomes a significant expenditure of time. For example, the interrogation of _10_C _2_ = 45 biaxial plots is required for the evaluation of 10 biomarkers. In addition, while robust, the inherent subjectivity of manual gating diminished its practical application for the clinical evaluation of specimens. Therefore, efficient and objective interpretation of mIHC-stained image entails several challenges limiting broad methodologic and clinical dissemination.

**Figure 6:**
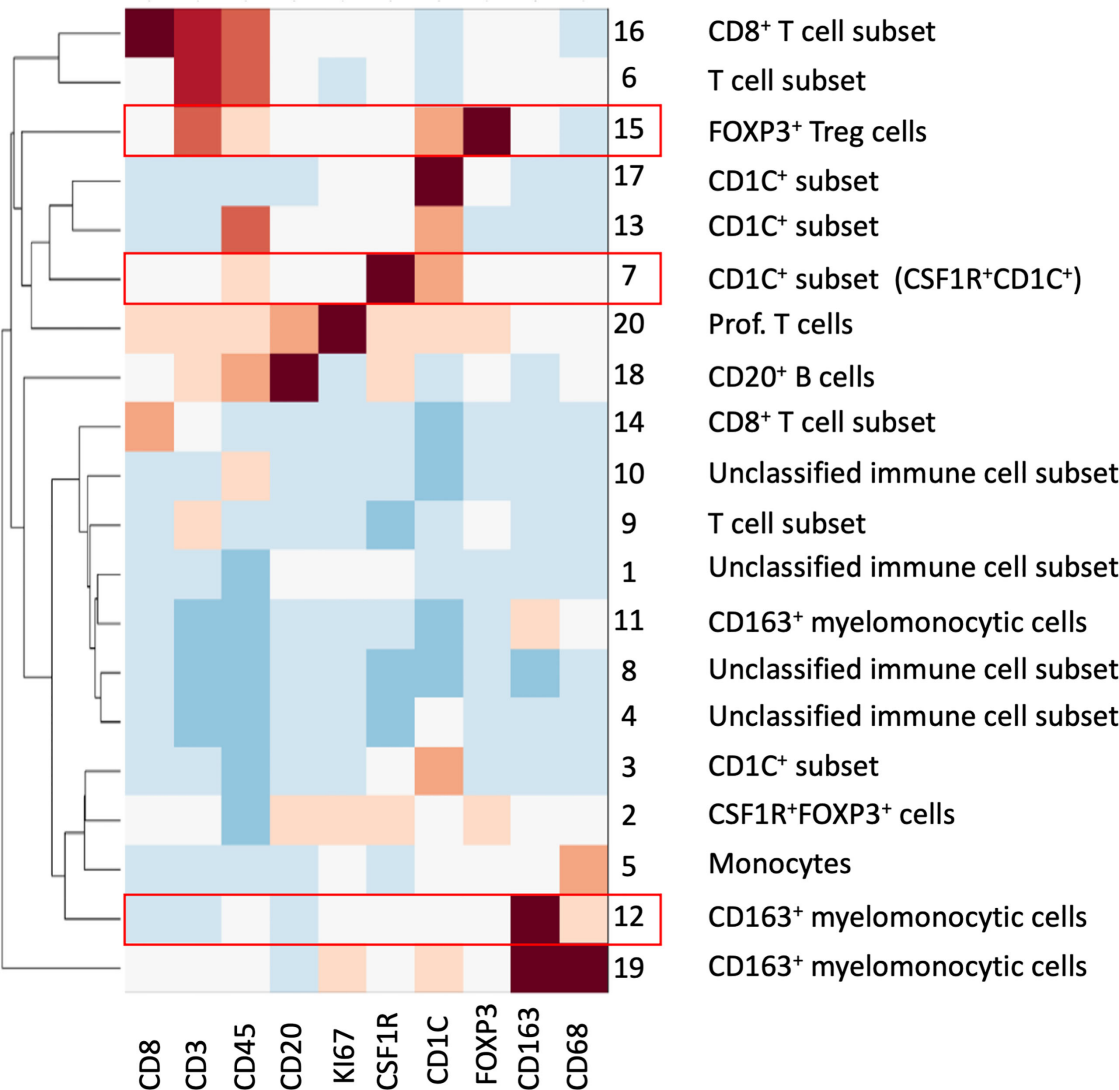
SSC of all immune cells from all ROIs based on the 10 mIHC markers. A total of 20 cell groups were interpreted based on their expression of mIHC markers. Cell groups with statistical significance were highlighted in red; please see also **Figure 7**.

To overcome the limitation of the manual gating strategy and handle both biological heterogeneity (e.g., various cell types) and high redundancy in feature representation, we adopted the SSC approach by extending a similar concept of sparse coding used in our previous work (22) (see *Materials and Methods*). The proposed SSC approach enables objective and automated cell clustering *via* a simultaneous assessment of all 10 markers from all ROIs. In addition, the SSC approach has a better overall performance than other approaches such as principal component analysis (PCA) when clustering data from incomplete observations, which is usually an issue in multiplex imaging data that not all features are available for every data point (30). Since we chose to focus on immune complexities here, we analyzed only immune cells by using a cut-off value for CD45 (>0.07 of mean marker intensity, based on manual gating image cytometry), the pan-immune cell maker. We extracted 78,769 CD45^+^ cells from the 40 ROIs and clustered into 20 groups based on the single-cell mean intensity of 10 markers (**Figure 6**). For our analysis, we simply explored a different number of groups, i.e., the number of subspace *k* (see *Materials and Methods*) to identify a smaller but distinct source of variation in the data with biological interpretation based on the elbow method. In general, the clustering results were comparable to the manual gating results and identified the key cell subsets including CD8^+^ T cells, FOXP3^+^ Treg cells, other T cell subsets, B cells, monocytes, and CD163^+^ myelomonocytic cells. This confirmed that our unsupervised analysis approach *via* SSC corroborated and complemented the manual gating strategy, which allowed the discovery of novel cell types and robust cell identification with high efficiency and objectivity from a large-scale singe-cell dataset.

**Figure 7:**
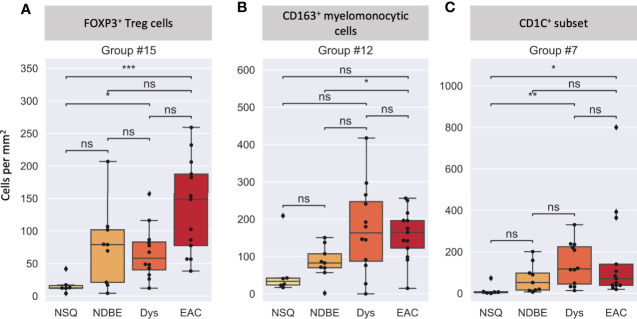
Cell density per mm2 at different disease stages of selected cell groups that clustered by SSC. **(A)** Group #15 was interpreted as FOXP3+ Treg cells, **(B)** Group #12 was interpreted as a CD163+ myelomonocytic cells (CD163^+^CD68^+^), and **(C)** Group #7 was interpreted as a new CD1C+ subset (CSF1R^+^CD1C^+^), please also see **Figure 6**. Each datapoint represent one ROI. Statistics: Mann-Whitney-Wilcoxon test two-sided with Bonferroni correction: ns (not significant): p > 0.05; *p ≤ 0.05; **p ≤ 0.01, ***p ≤ 0.001.

We then looked at the cell density of the SSC-clustered cell groups in the disease progression (**Figure 7**, **S8**). It was noteworthy that the immune cell populations with significant change in disease progression, Treg cells and the CD163^+^ myelomonocytic cell subset, were identified using the SSC approach as group #15 (**Figures 6**, **7A**) and #12 (**Figures 6**, **7B**), respectively, and showed the similar change. Interestingly, the SSC approach also identified a myeloid cell lineage; group #7 characterized by CSF1R and CD1C (**Figure 6**), which was not a predefined population in the manual gating strategy, also showed significant increase with disease progression (**Figure 7C**).

We reasoned that the SSC approach clustered some immune populations into distinct subsets that were not pre-defined by image cytometry manual gating. For example, groups #11, #12, and #19 were all attributed to CD163^+^ myelomonocytic cells based on the high staining density of CD163, but only group #12 was found to have a significant increase that showed the same trend in manually gated CD163^+^ myelomonocytic cells (**Figure 4C**). In addition, the cell densities of the SCC-clustered CD163^+^ myelomonocytic cell subset (Group #12) in NSQ, NDBE, Dys, and EAC (**Figure 7B**) were all proportional to the manually gated CD163^+^ myelomonocytic cells (**Figure 4C**). We therefore cautiously concluded that this specific myelomonocytic cell subset (Group #12) might be a subset of total CD163^+^ myelomonocytic cells, which is more likely to play a role in the progression of the disease compared with the other subsets. We postulated the same for the new CD1C^+^ subset (CSF1R^+^CD1C^+^, group #7) belonging to a wider CD1C^+^ population in manual gating. There were four subsets: Groups #1, #4, #8, and #10, which all showed relatively low intensities of most markers. They were labeled as unclassified immune cells and are elaborated in *Discussion*.

### Spatial Analysis of SSC-Identified CD163, CD1C Subsets, and Treg Cells in Disease Progression

**Figure 8:**
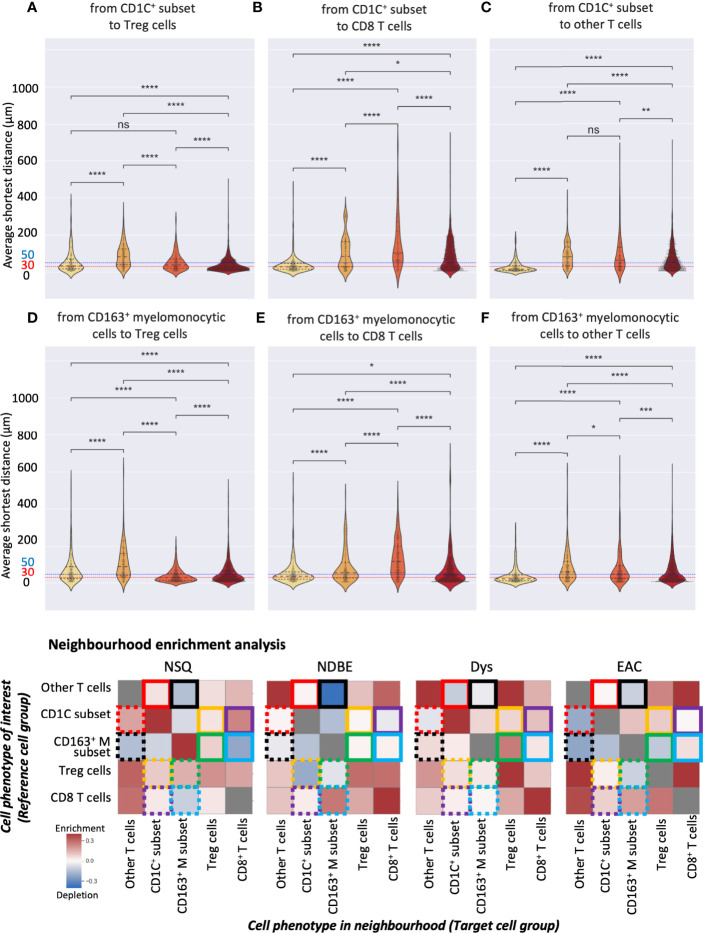
Spatial analysis of selected SSC cell subsets. **(A–F)** Average distance analysis: violin plots represented the shortest average distance from one cell subset to another. Statistics: Mann–Whitney–Wilcoxon test two-sided with Bonferroni correction: ns (not significant): p > 0.05; *: p <=0.05; **: p <=0.01, ***: p <=0.001, ****: p <=0.0001. **(B)** Neighborhood enrichment analysis: the heat map represents the enrichment or depletion of a cell group in the neighborhood of another; colored boxes denote pairs of cell subsets, e.g., yellow boxes denote the analysis of the CD1C^+^ subset and Treg cells; each box represents the enrichment/depletion of the target cell group in the neighborhood of the reference cell group or cell phenotype of interest; gray boxes indicate enrichment/depletion that is not significant.

Both manual gating and SSC approaches revealed a clear increasing density of Treg cells, the CD163^+^ myelomonocytic cells, and the new CD1C^+^ cell subset. We then further studied their spatial relationship for possible cell-cell interactions during disease progression by interrogating the SSC data using two spatial analyses. First, we calculated the average shortest distance between the centers of two cell nuclei of two given cell subsets and applied 30 and 50 μm as two thresholds to evaluate cell proximity (**Figures 8A–F**, **S9A**). Although reflecting the absolute distance between cells, the average shortest distance could be affected by the overall cell compactness and distribution pattern. We therefore applied a second approach of neighborhood enrichment analysis that computed the likelihood that a given cell subset was neighbored by other cell subsets compared to the random background (**Figures 8G**, **S9B**).

We first focused on the CD1C^+^ subset and examined their neighbors. The shortest average distance observed from the CD1C^+^ subset to Treg cells followed a clear gradual decrease from NDBE to Dys and then EAC (**Figure 8A**). Especially in EAC, the majority of the cells of the two subsets were within 30 μm. Neighborhood enrichment analysis also revealed that Treg and CD1C^+^ subsets were slightly depleted in each other’s neighborhood in NDBE but significantly enriched in Dys and EAC (**Figure 8G**, highlighted by yellow solid- and dotted-line boxes). A similar finding was observed in the average distance from the CD1C^+^ subset to CD8^+^ T cells, but it was generally further (>50 μm) (**Figure 8B**) compared with the distance from CD1C^+^ to Treg cells through all disease stages (**Figure 8A**). In the neighborhood of CD8^+^ T cells, there were increasingly enriched CD1C^+^ cells from NDBE to Dys and EAC (**Figure 8G**, highlighted by purple dashed-line boxes); but in the neighborhood of CD1C^+^ subsets, the enrichment of CD8^+^ T cells varied between the disease stages (**Figure 8G**, highlighted by purple solid-line boxes). The CD1C^+^ subset was unlikely to have direct cell–cell interaction with other T cells in all disease stages because they were not enriched in each other’s neighborhoods and the average distances were far (**Figures 8C, G**, highlighted by red solid- and dotted-line boxes).

We then focused on the CD163^+^ myelomonocytic cell subset. The shortest average distance (<30 μm) was observed from CD163^+^ myelomonocytic cells to Treg in Dys (**Figure 8D**), which also had the most significant neighborhood enrichment (**Figure 8G**, highlighted by green solid-line boxes in Dys). The shift of their neighborhood phenotype from a slight depletion in NDBE to enrichment in Dys (**Figure 8G**, highlighted by green solid-line boxes, NDBE vs. Dys), mirrored the shift of the proportion of CD163^+^ myelomonocytic cells, which increased from 15% to 45% (**Figure 5B**, NDBE vs Dys). This was consistent with literature reporting that Treg cells induce the polarization of CD163^+^ myelomonocytic cells (referred to as M2-like macrophages in the literatures) (31–33). In EAC, the average distance from CD163^+^ myelomonocytic cells to Treg cells was close (**Figure 8D**, EAC), but it was unlikely that they were spatially correlated, as revealed by the neighborhood enrichment analysis (**Figure 8G**, highlighted by green solid- and dotted-line boxes, EAC). This was likely due to Treg cells having significantly higher density in EAC (**Figures 4B** and **7A**), such that they were spatially closer to all their neighbors but not specifically to the CD163^+^ myelomonocytic cell subset. The average distance from CD163^+^ myelomonocytic cells to CD8^+^ T cells was greater overall (**Figure 8E**) than to Treg cells (**Figure 8D**). CD8^+^ T cells were increasingly enriched in the neighborhood of CD163^+^ myelomonocytic cells in disease progression (**Figure 8G**, highlighted by blue solid-line boxes), but variation was observed in the vice versa (**Figure 8G**, highlighted by blue dotted-line boxes). The spatial relationship between CD163^+^ myelomonocytic cells and other T-cell subsets was unclear, where the average distance decreased with disease progression (**Figure 8F**), but the neighborhood phenotype varied (**Figure 8G**, highlighted by black solid- and dotted-line boxes). It was not unexpected as this T-cell group was probably a diverse population consisting of multiple T helper cell subsets.

## Discussion

In this study, we modified our previously described mIHC methodology with an automated staining protocol and developed image analysis pipelines, including nuclear segmentation, automated image co-registration, and an unsupervised clustering approach based on SSC for cell phenotyping. The pipelines were demonstrated to be effective and accurate when acquiring our dataset under the 10 immune lineage biomarkers. An unsupervised clustering approach *via* SSC successfully identified the cell populations with statistically significant changes and further identified the CD1C^+^ myeloid subset (CSF1R^+^CD1C^+^), highlighting its potential in processing mega-scale mIHC datasets and discovering unique cell populations.

Using complement analytic approaches of image cytometry manual gating and unsupervised SSC, and two spatial analytical approaches of the average shortest distance and neighborhood enrichment analysis, we characterized the immune complexity in the stepwise normal-metaplasia–Dys-malignancy progression on well-defined ROIs of all disease stages. To summarize, in the first step from NSQ to NDBE, the total immune cell density significantly increased, specifically the Treg cells and CD1C^+^ subsets. From NDBE to Dys, total immune cell and Treg cell densities stabilized, while the CD1C^+^ subset and CD163^+^ myelomonocytic cells continued to show increasing trends and became more proximal with Treg cells. Notably, only at this stage, the proportion of CD163^+^ myelomonocytic cells in the total myelomonocytic cells significantly increased. At the last neoplastic stage from Dys to EAC, total immune cells and Treg cells significantly increased in density again while the other immune cells largely remained unchanged. The study also shed a light on the possible mechanism of T-cell subset recruitment and polarization from BE to EAC, where the interaction between the new CD1C^+^ subset and Treg cells appeared to increase during the progression of disease, indicating their potential role in immunosuppression during EAC development.

An understanding of the complex immune populations in cancer has been increasingly important in the past decades and has been studied under various topics including cancer initiation, progression, metastasis, prognosis, and efficacy of immunotherapy (34–36). EAC serves as an excellent model for cancer initiation and progression because it can be clearly categorized into histopathologically defined stages such that the immune complexity can be studied at each individual stage. However, it is only recently that multi-parametric methods have begun to emerge. To our knowledge, there has been only one other study aiming to comprehensively depict the immune landscape in the BE and EAC progression: in 2021, Lagisetty and colleagues used xCell, an algorithm that deconvolutes bulk RNA-seq data, to determine the prominent immune components in BE, Dys, and EAC (37). Consistent with our observations, the authors also found increased presence of Treg cells and CD163^+^ myelomonocytic cells (referred to as M2-like macrophages in the study) in disease progression. However, xCell revealed that the increase of CD163^+^ myelomonocytic cells occurred between high-grade Dys and EAC, whereas it was not significant in our results. This discrepancy may be attributed to different types of raw data, RNA-seq compared with mIHC, and thus does not necessarily have highly expected concordance. It is noteworthy that the 2021 Lagisetty study also validated the xCell results using a commercially available 6-marker multiplex method for CD3, CD8, CD163, FOXP3, PD-L1, and PanCK. Only the IHC, but not xCell, identified an abrupt decrease in FOXP3^+^ Treg cells between Dys and EAC, whereas our 10-marker mIHC found the opposite: that the Tregs significantly increased. Interestingly, our results on Treg cells were actually in line with the overall conclusion of the 2021 Lagisetty study that EAC reflects an immunosuppressive microenvironment. We reasoned that the contradictory observation on Treg cells might be due to the fact that the 2021 Lagisetty study used only the tissue cores from tissue microarrays (TMAs) that have limited stromal components, while we used full-thickness EMR tissue and specifically included the stromal margin adjacent to the epithelium where immune cell traffic is commonly found. In addition, we also combined low- and high-Dys into a single stage and only used EAC with the T1a stage, whereas in the Lagisetty study, low- and high-Dys were studied separately, and the EAC samples ranged from stage 1 to 3.

It was interesting that both this study and ours identified CD163^+^ myelomonocytic cells in the disease progression, although different methods were used. CD163 has been commonly used as a marker for the M2-like type of tumor-associated macrophages (TAMs), which include a range of diverse cell populations that share an overall immunosuppressive function (38, 39). How such function is exerted still attracts active research. For example, it has been reported that M2-like TAMs express a high level of IL-10 (40), an immunosuppressive cytokine that suppresses antigen-presenting cell function (41), while maintaining the Treg cell function, which could also be recruited by M2-like TAMs, leading to a positive feedback loop of immunosuppression (42–44). Although the molecular mechanism remains not entirely clear, M2-like TAMs have been associated with the general poor prognosis and aggressiveness of many cancer types [reviewed in (45)], including breast cancer (46), liver cancer (47), and non-small-cell lung cancer (48). It is noteworthy that the identification of M2-like macrophages in clinical samples usually relied on the immunostaining of one or two markers, which are unlikely to be sufficient to mark a specific macrophage population with a sole phenotype. In this study, we used a more general term, “CD163^+^ myelomonocytic cells,” which was mainly identified by its high expression of CD163 and may include other cell types such as monocytes. Our discovery here highlighted the importance of using techniques that allow for more markers to dissect the complex cell composition, identify specific immune cell subsets, and take spatial context into consideration when studying patient samples.

Together, these innovations and research findings highlight several key factors in the rapidly developing multiplex imaging field (summarized in **Table 4**). First, in addition to the choice of tissues, accurate detection of tissue-based biomarkers is essential, typically achieved by immunostaining in most multiplex methods. Although antibodies are widely available for such research purposes, their performance should be validated and confirmed by pathologists, especially when used in clinical samples. mIHC is advantaged in this regard as pathologists are more familiar with evaluating tissue staining based on peroxidase colorimetric IHC approaches. Second, depending on how images are acquired, current multiplex methods can be categorized into simultaneous and sequential. The former acquires the images of all markers in one round, such as routine multi-color immunofluorescence or Image CyTOF, whereas the latter relies on sequential cycles of detection and removal of each marker, thus requiring a robust approach to coregister images. Coregistration could be challenging because images are likely to be acquired days apart and the tissues are subjected to repeated cycles of heating and washing to remove the previous markers. In this study, we demonstrated that the feature matching-based algorithm was extremely effective and accurate for mIHC image coregistration. Third, all multiplex imaging methods quantify marker expression through the signal deconvolution of corresponding images, which is digitally achieved, involving three major steps: nuclear segmentation that recognizes the cell nucleus and marks the boundary of the cell; deconvolution of raw images to isolate signal intensities that are measured within the boundary of the cell; and the conversion of the signal intensity into a single value that represents the marker expression level. In general, methods with higher image resolution will lead to more accurate quantification and allow for more sophisticated computation algorithms (55). Microscopic-based imaging methods, such as mIHC, CyCIF (53), and CODEX (54) yield higher-resolution images compared with mass spectroscopy-based Image CyTOF (51). Fourth, a marker’s staining intensity does not directly represent the protein level in a single cell, providing only a slice of the cell, rather than an intact cell, being stained. This is a universal issue to all two-dimensional multiplex imaging methods and imperfect segmentation labels. For example, in flow cytometry analysis that examines intact cells, CD3^-^CD8^-^, CD3^+^CD8^+^, and CD3^+^CD8^-^ populations are represented by fully separated scatters, whereas in image-based methods, the populations are less separated and have small ambiguous overlaps (**Figure S4D** in (20) This may hinder the objectivity and efficiency when interpreting high-dimensional multiplex imaging data. To overcome these issues, we applied the SSC approach based on the extension of our previous work (22) and demonstrated its efficiency and objectivity in analyzing mIHC data.

**Table 4:** Summary of multiplex imaging methods.

	Classic IHC or IF	Improved/Commercial multiplex IF	Image Mass Cytometry (Image CyTOF)	Slide-Seq	Sequential Multiplex IHC	CyCIF	CODEX
No. of markers	1–3 markers	Up to 6 markers	~30 markers	n/a (RNA based)	10–20 markers	10–20 markers	10–30 markers
Quantitative	Semi-quantitative	Semi-quantitative	Semi-quantitative	Quantitative	Semi-quantitative	Semi-quantitative	Semi-quantitative
Tissue required	A single FFPE	A single FFPE	A single FFPE	Freshly sectioned cryo tissue	A single FFPE	A single FFPE	A single FFPE
Image resolution	High (0.2–0.5 µm per pixel)	High (0.2–0.5 µm per pixel)	Medium (1 µm per pixel)	Low and not cell based (10 µm per pixel/bead)	High (0.2–0.5 µm per pixel)	High (0.2–0.5 µm per pixel)	High (0.2–0.5 µm per pixel)
Image acquisition	Simultaneous	Simultaneous	Simultaneous	Simultaneous	Sequential	Sequential	Sequential
Specialized antibodies needed?	No	Yes Commercial kit Primary antibodies with specific fluorophores	Yes Commercial kit Primary antibodies with specific metal isotopes	No But need specialized beads	No	No	Yes Primary and secondary antibodies with specific oligos
Specialized equipment for imaging needed?	No	Yes Commercial equipment (Vectra, etc, multi-color fluorescent scanner)	Yes Commercial equipment (Hyperion Fluidigm)	No But need sequencer	No Common brightfield slider scanner	Maybe Fluorescent slider scanner	Yes Commercial equipment for staining and image acquisitions
Cost	Low	High	Very high	High–very high	Medium	Medium	Medium–high
Reference	(49)	(50)	(51)	(52)	(20)	(53)	(54)

The increasing application of multiplex imaging techniques also leads to a rapid development of image analysis tools, such as SSC in this study. Apart from the efficiency and accuracy, it is possible that these unsupervised clustering tools will generate subsets that, if biologically relevant, are difficult to interpret for their identities. For example, unclassified immune cell subsets in this study and “CD45^+^ other” in other multiplex imaging studies (56). It does not necessarily mean that these cells do not have biological significance, but they are unclear if analyzed using only one clustering method and a limited antibody panel. We therefore applied two approaches herein. In spatial analysis, we also observed that the average distance between all cell subsets was overall closer in a normal esophagus. We postulated that that was related to the distinct microarchitecture of the squamous epithelium of a normal esophagus, as compared with the columnar epithelium of NDBE, Dys, and EAC. A squamous epithelium is known for an undulating pattern of rete ridges that a columnar epithelium lacks; immune cells were more enriched at sites with higher microvascular densities along rete ridges (57), which were also observed in a normal esophagus (**Figure S10**). It is also important to distinguish between reference and target subsets in spatial analysis. For example, in Dys, CD163^+^ myelomonocytic cell subsets were enriched in Treg cell neighborhoods, but Treg cells were not abundant in CD163^+^ myelomonocytic cell neighborhoods.

There were limitations in this study in that our cohort was relatively small, and all the antibodies used for panel design were targeted to discrete immune cell lineages, and epithelial and stromal cells were not studied. However, we compensated with well-annotated ROIs and a deep interrogation of the data using multiple independent analytical approaches. Our results shed light on the dynamic changes in the immune landscapes during the development and progression of BE and EAC and highlighted a few potential candidate cell types of interest, including CD1C^+^ and CD163^+^ cell subsets. Taken together, this study points to a number of future research directions for deciphering complex cell phenotypes and interactions using minimal clinical samples, opening up new avenues for translational research in cancer. Conclusions and methods in this study paved the path to more effective strategies for early detection of EAC and risk stratification of BE patients, and continued efforts are needed to further investigate the precise roles of the various immune populations discovered in this study and validate the biomarkers in larger patient cohorts.

## Data Availability Statement

The original contributions presented in the study are included in the article/**Supplementary Material**. Further inquiries can be directed to the corresponding authors.

## Ethics Statement

The studies involving human participants were reviewed and approved by East of England - Cambridge Central Research Ethics Committee. The patients/participants provided their written informed consent to participate in this study.

## Author Contributions

SSu performed the mIHC experiments and data analysis and prepared the initial report and figures excluding SSC results and spatial analysis. EK performed data analysis and prepared the manuscript and figures. GJ performed and optimized the mIHC method. SSi established the computational pipeline of co-registration. MT and AM assessed the EMR tissue and graded the local pathological grades. MP supervised the collection of the endoscopic samples. LC and RF conceived the study design and analysis and provided guidance on preparing the manuscript. YC and LZ conceived the study design and analysis, performed the analysis, supervised the research, and prepared the manuscript and figures.

## Funding

This work was supported by the CRUK-OHSU joint grant to YC and LZ (C65718/A29808) and was supported in part by the National Cancer Institute - U54CA209988. YC and LC acknowledges funding from the National Institutes of Health (1U01 CA224012, U2C CA233280), the Knight Cancer Institute, and the OHSU-Brenden-Colson Center for Pancreatic Care. The laboratory of RF is funded by a Core Programme Grant from the Medical Research Council (RG84369).

## Author Disclaimer

The views expressed are those of the authors and not necessarily those of the NIHR or the Department of Health and Social Care.

## Conflict of Interest

LC reports consulting services for Cell Signaling Technologies, AbbVie, the Susan G Komen Foundation, and Shasqi, received reagent and/or research support from Cell Signaling Technologies, Syndax Pharmaceuticals, and Acerta Pharma, and has participated in advisory boards for Pharmacyclics, Syndax, Carisma, Verseau, CytomX, Kineta, Hibercell, Cell Signaling Technologies, Alkermes, Zymeworks, the AstraZeneca Partner of Choice Network, the Lustgarten Foundation, and the NIH/NCI-Frederick National Laboratory Advisory Committee. RF holds patents related to Cytosponge-TFF3 and related assays that have been licensed by the Medical Research Council to Covidien (now Medtronic). RF is a co-founder and shareholder in an early detection and digital pathology company Cyted Ltd.

The remaining authors declare that the research was conducted in the absence of any commercial or financial relationships that could be construed as a potential conflict of interest.

## Publisher’s Note

All claims expressed in this article are solely those of the authors and do not necessarily represent those of their affiliated organizations, or those of the publisher, the editors and the reviewers. Any product that may be evaluated in this article, or claim that may be made by its manufacturer, is not guaranteed or endorsed by the publisher.
