# Supplementary material for: Deciphering the Immune Complexity in Esophageal Adenocarcinoma and Pre-Cancerous Lesions With Sequential Multiplex Immunohistochemistry and Sparse Subspace Clustering Approach
Source: Front Immunol. 2022 May 19;13:874255. doi: 10.3389/fimmu.2022.874255 (PMC9161782; doi:10.3389/fimmu.2022.874255)
Supplement: Supplementary file 1 [file DataSheet_1.pdf]

Supplementary figures

Figure S1

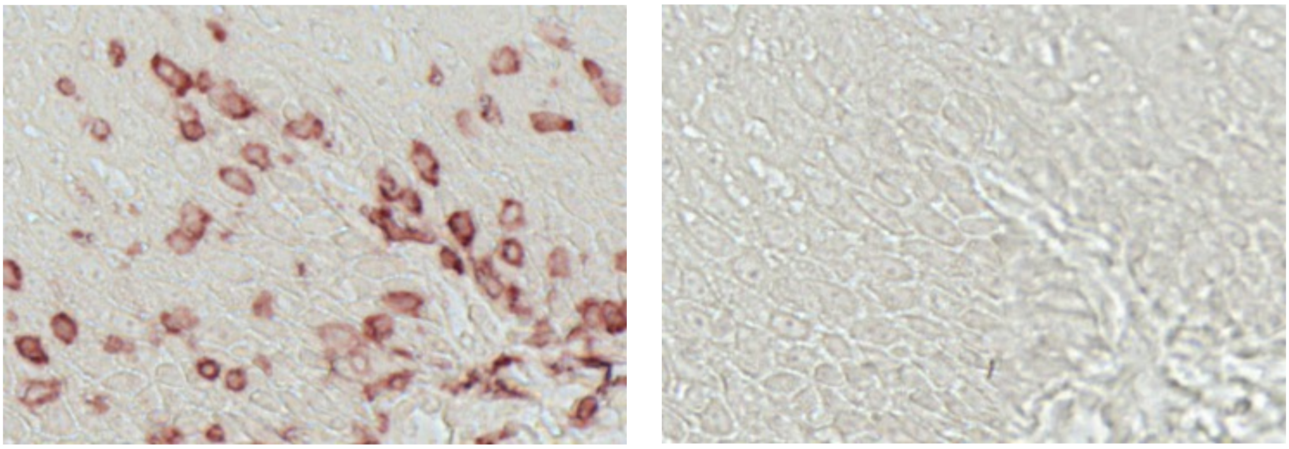

CD45 in Cycle 1 CD20 in Cycle 2

Figure S1. Two cycles of mIHC staining of CD45 and CD20. Note the complete removal of CD45 staining in the next cycle. This region was stained negative for CD20, too.

Supplementary figures

Figure S2

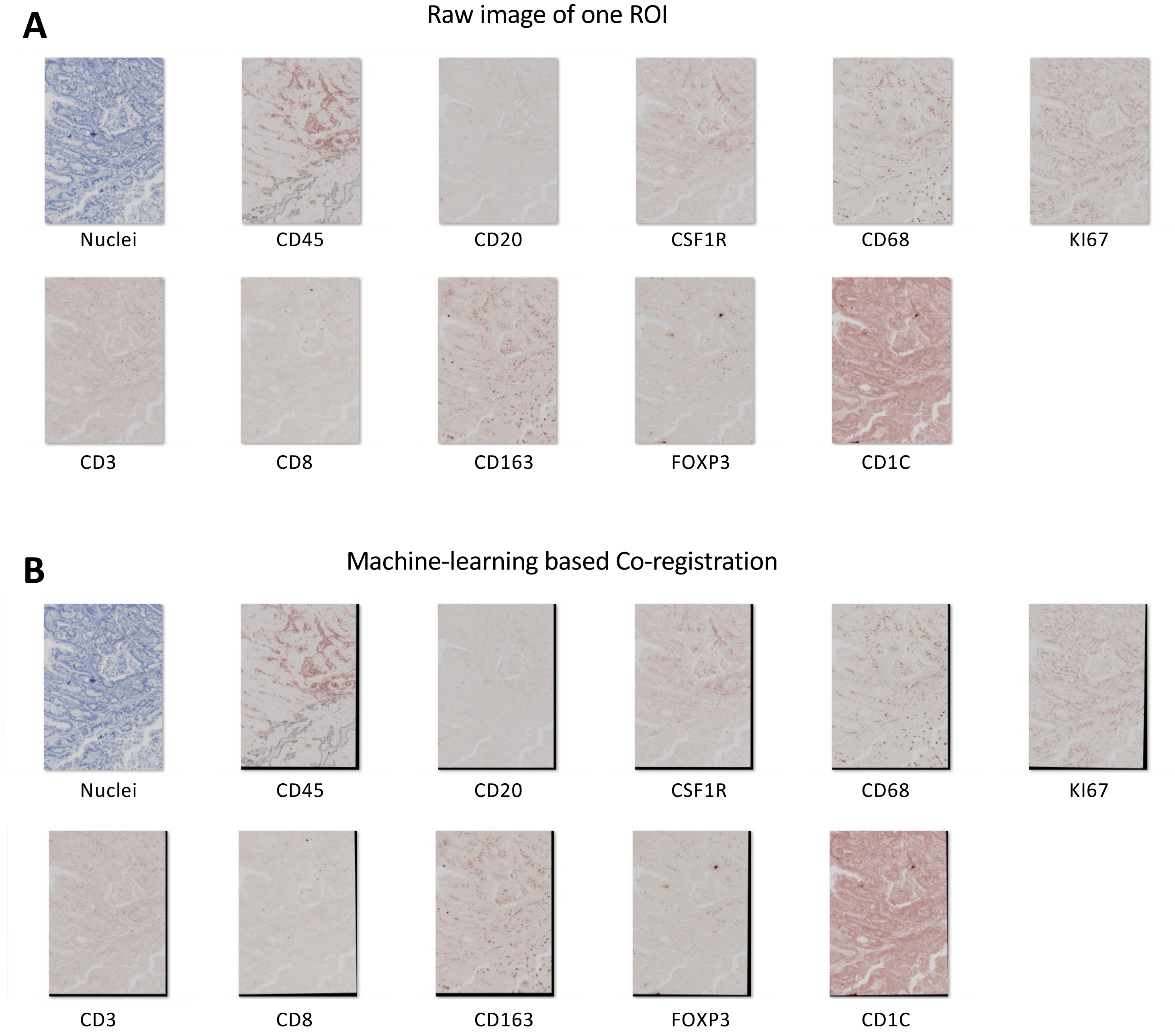

Figure S2. (A) Raw images of one ROI that respond to Nuclei and all 10 markers; (B) Co-registered images of (A) using machine-learning based approach. Note the black lines around the co-registered images, indicating the image was 'moved' to register to the Nuclei image.

Supplementary figures

Figure S3

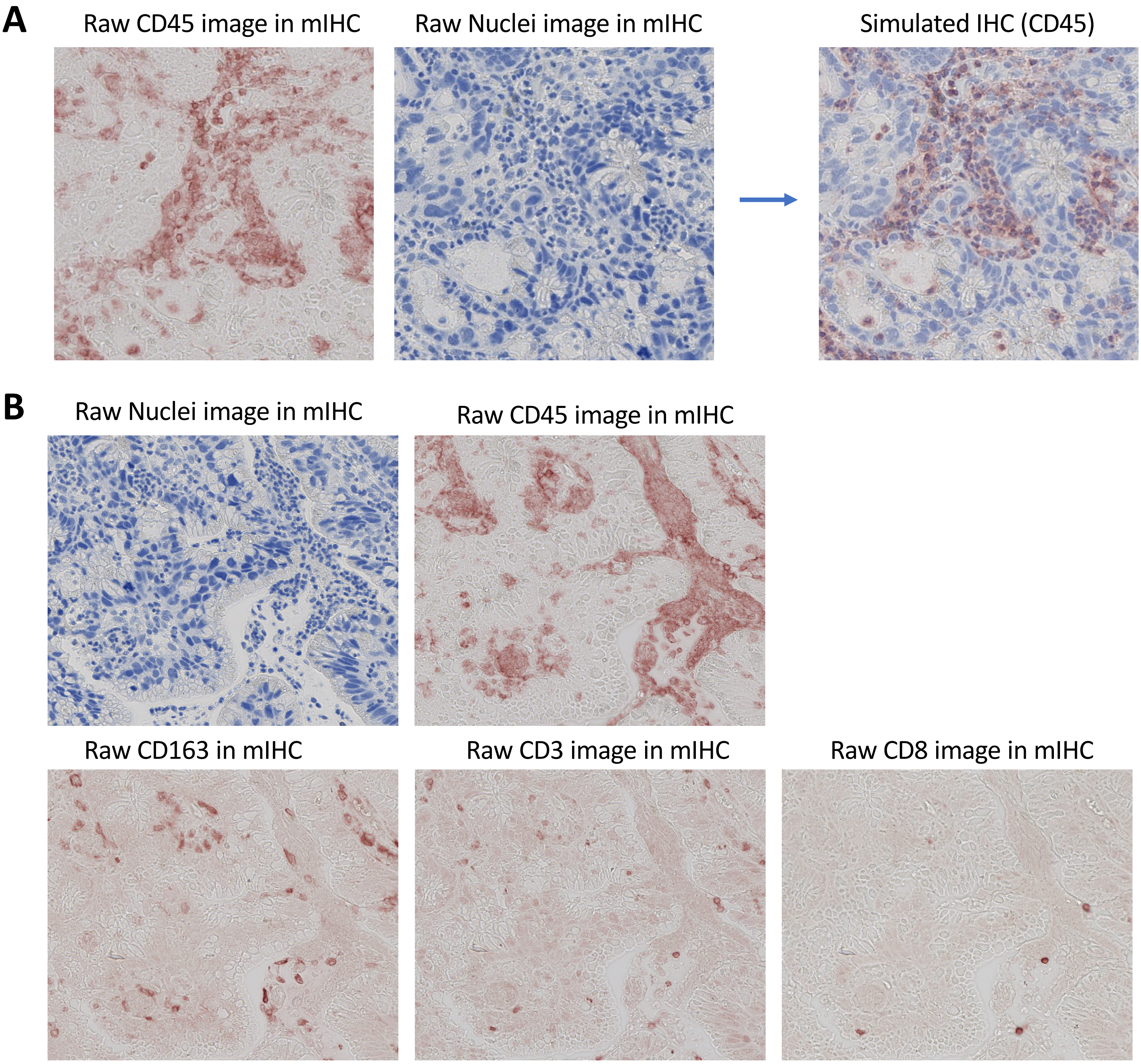

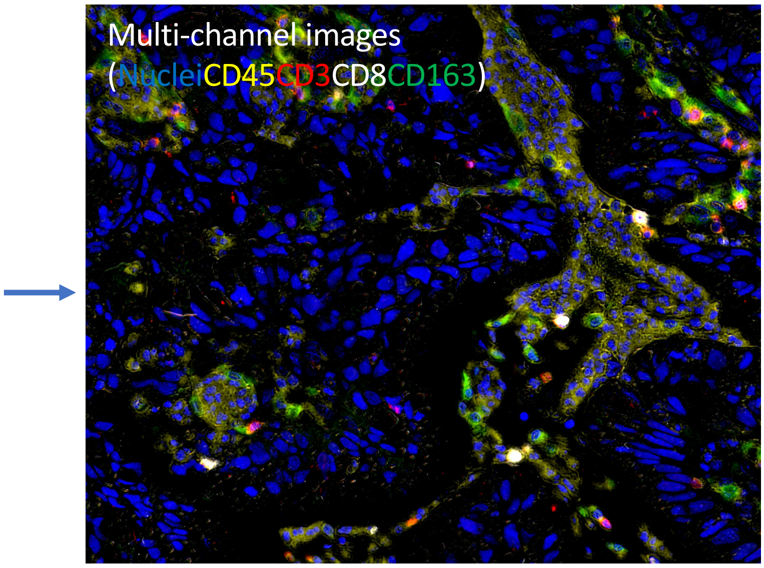

Figure S3. (A) multi-channel merged IHC image (CD45) digitally generated by raw images of CD45 and Nuclei in mIHC, readily to be assessed by pathologists. (B) Simulated multi-channel image (CD45/CD3/CD8/CD163) digitally generated by raw images of CD45, CD163, CD3, CD8 and Nuclei.

Supplementary figures

Figure S4

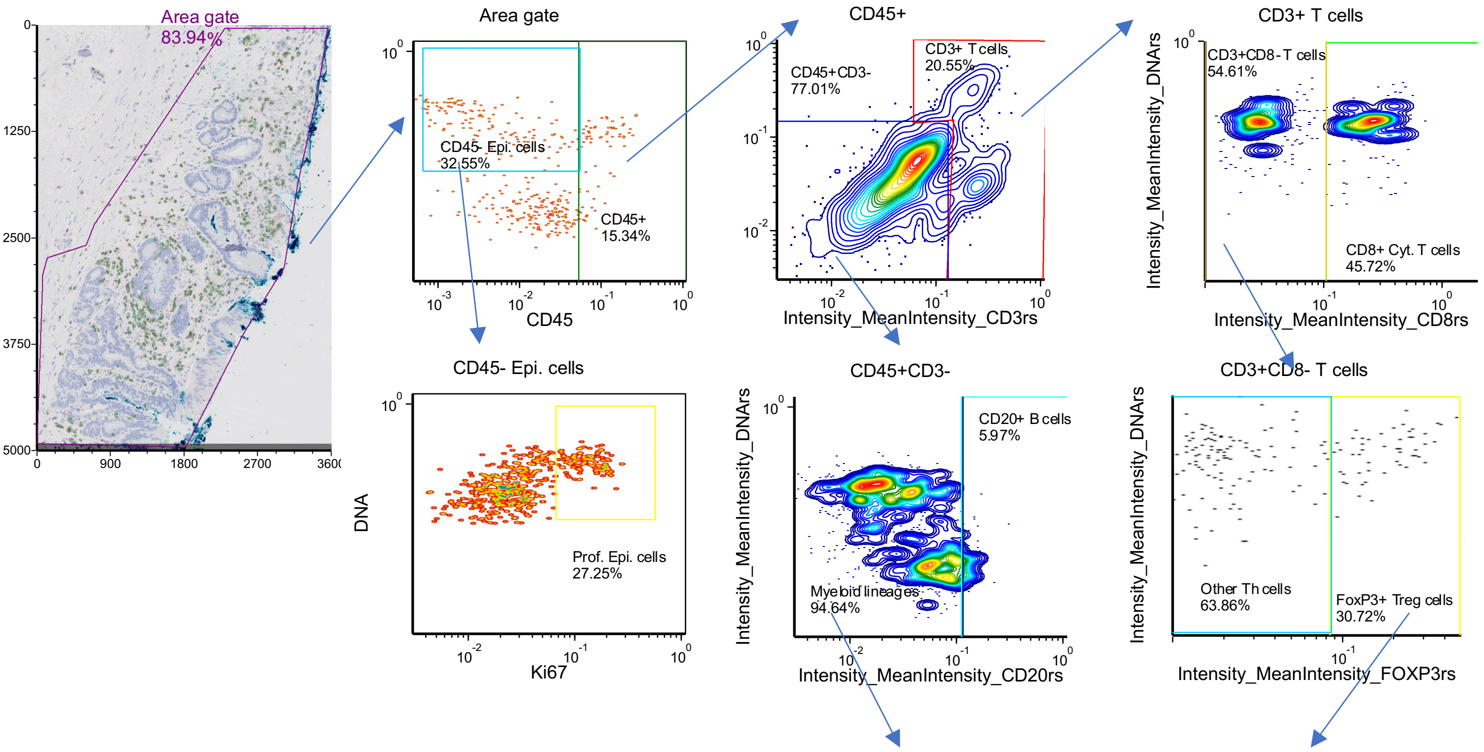

Gate	# of Events	% of gated cells	% of all cells
None	11487	100.00	100.00
Area gate	9642	83.94	83.94
CD45- Epi. cells	3138	27.32	27.32
Prof. Epi. cells	855	7.44	7.44
CD45+	1479	12.88	12.88
CD3+ T cells	304	2.65	2.65
CD8+ Cyt. T cells	139	1.21	1.21
Prof. Cyt. T cells	51	0.44	0.44
CD3+CD8- T cells	166	1.45	1.45
FoxP3+ Treg cells	51	0.44	0.44
Prof. Treg cells	3	0.03	0.03
Other Th cells	106	0.92	0.92
CD45+CD3-	1139	9.92	9.92
CD20+ B cells	68	0.59	0.59
Myeloid lineages	1078	9.38	9.38
CD1c+ mDCs	12	0.10	0.10
CD1c- Myeloid	1042	9.07	9.07
Macrophages	820	7.14	7.14
CD163+ M	243	2.12	2.12
CD163- M	566	4.93	4.93
Monocytes	112	0.98	0.98
CD68+	323	2.81	2.81
CD68+ CD163+	186	1.62	1.62
CSF1R+ CD68+	68	0.59	0.59
CD163+_v2	40	0.35	0.35
CD68+ CSF1R-	125	1.09	1.09

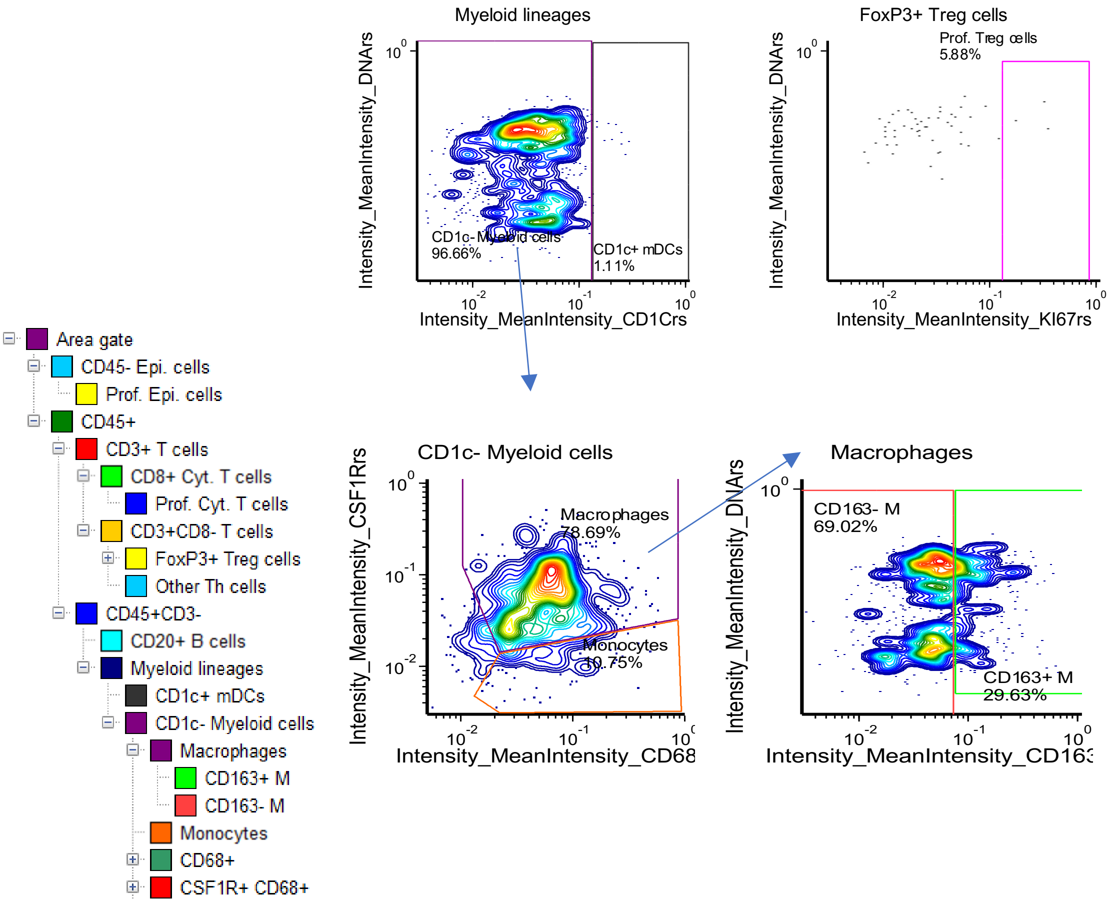

Figure S4. Gating strategy of various cell populations using Image Cytometry. Arrows denote gated cells presented in a new window for further gating. Note area gating could select cells direct from the ROI image.

Supplementary figures

Figure S5

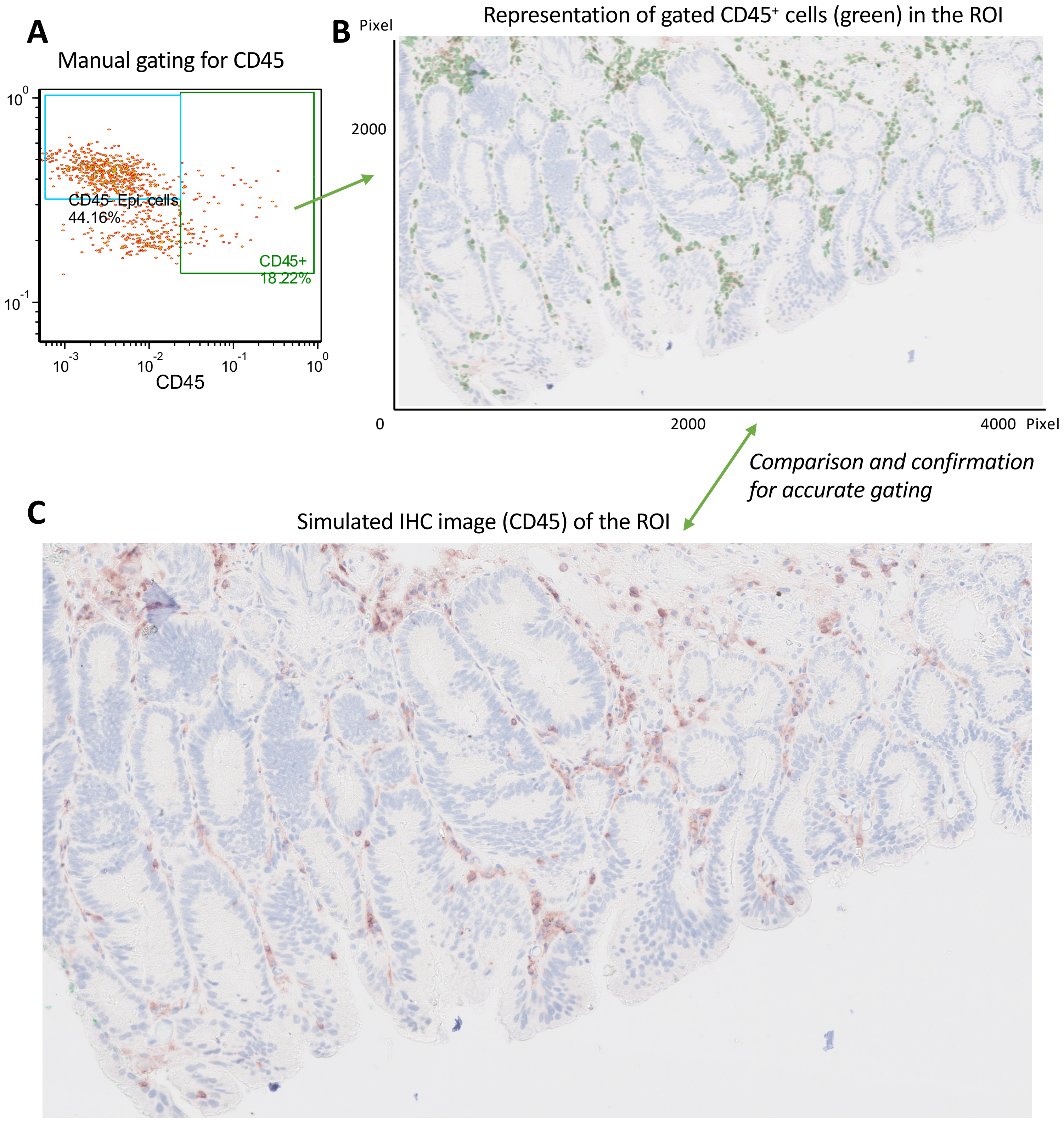

Figure S5. (A) Gating of CD45$^+$ cells in Image Cytometry; (B) gated CD45$^+$ cells in (A) were in highlighted in green in the original ROI; (C) simulated IHC image of CD45. Accurate CD45 gating was confirmed by check if all green cells correspond to the CD45 IHC and vice versa.

Supplementary figures

Figure S6

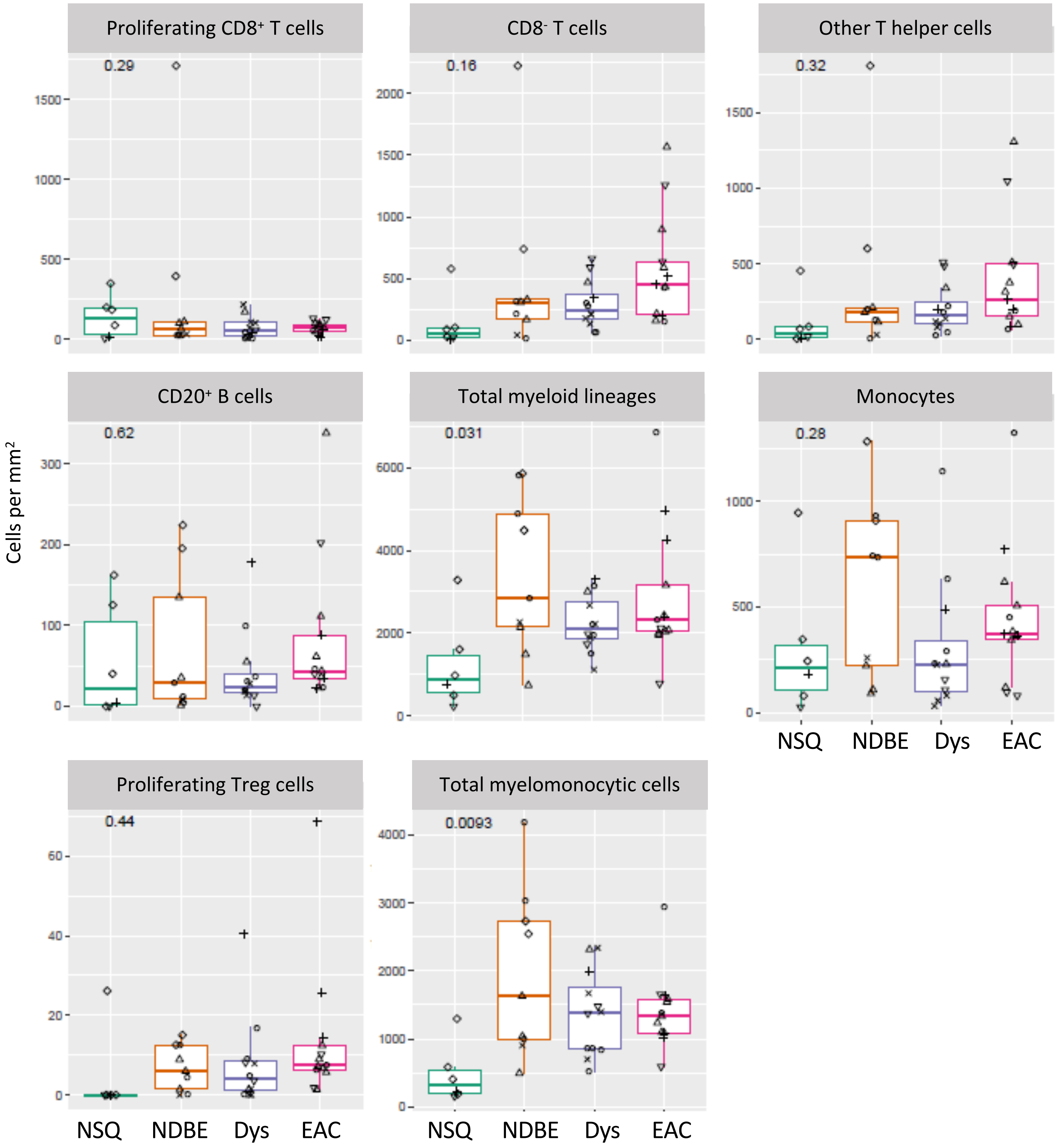

Figure S6. Cell density per mm^2 of various gated cell subsets based on Image Cytometry at different disease stage. Each datapoint represent one ROI. Statistics: Kruskal-Wallis One-Way ANOVA test.

Supplementary figures

Figure S7

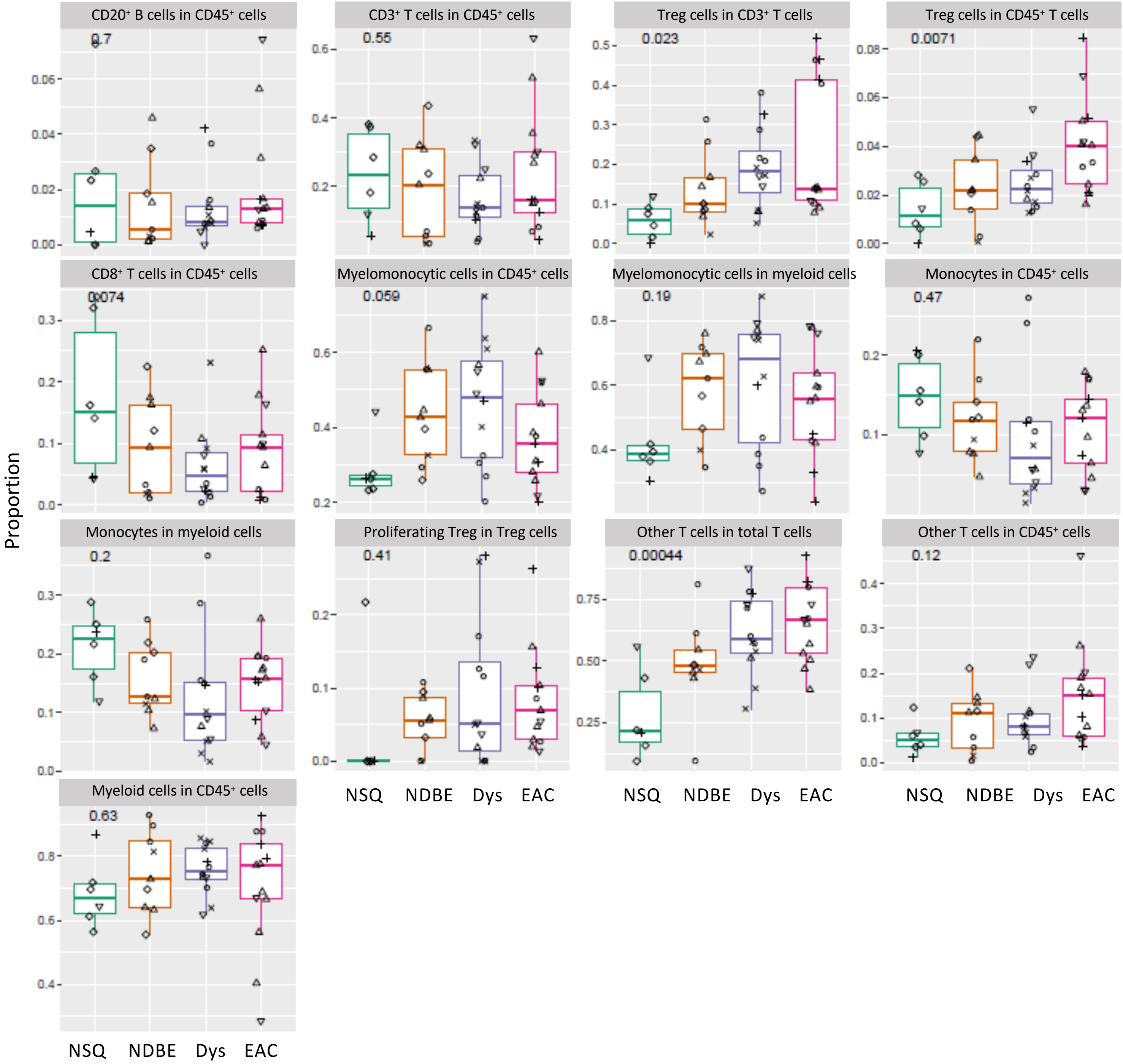

Figure S7. Cell proportion of various gated cell subsets based on Image Cytometry at different disease stage. Each datapoint represent one ROI. Statistics: Kruskal-Wallis One-Way ANOVA test.

Supplementary figures

Figure S8

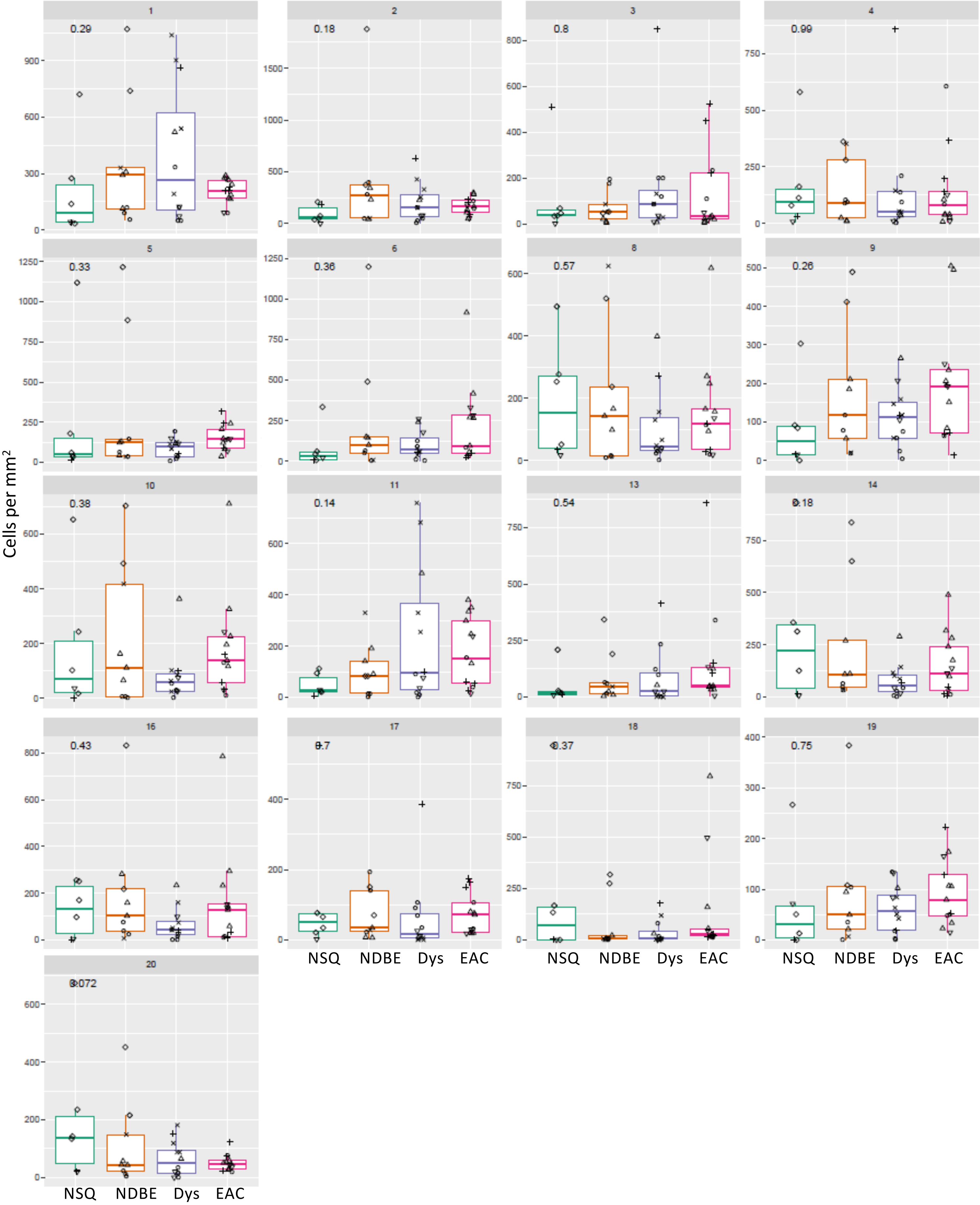

Figure S8. Cell density per mm^2 at different disease stages of cell groups that clustered by SSC, please also see Fig. 6. Each datapoint represent one ROI. Statistics: Kruskal-Wallis One-Way ANOVA test.

Supplementary figures

Figure S9

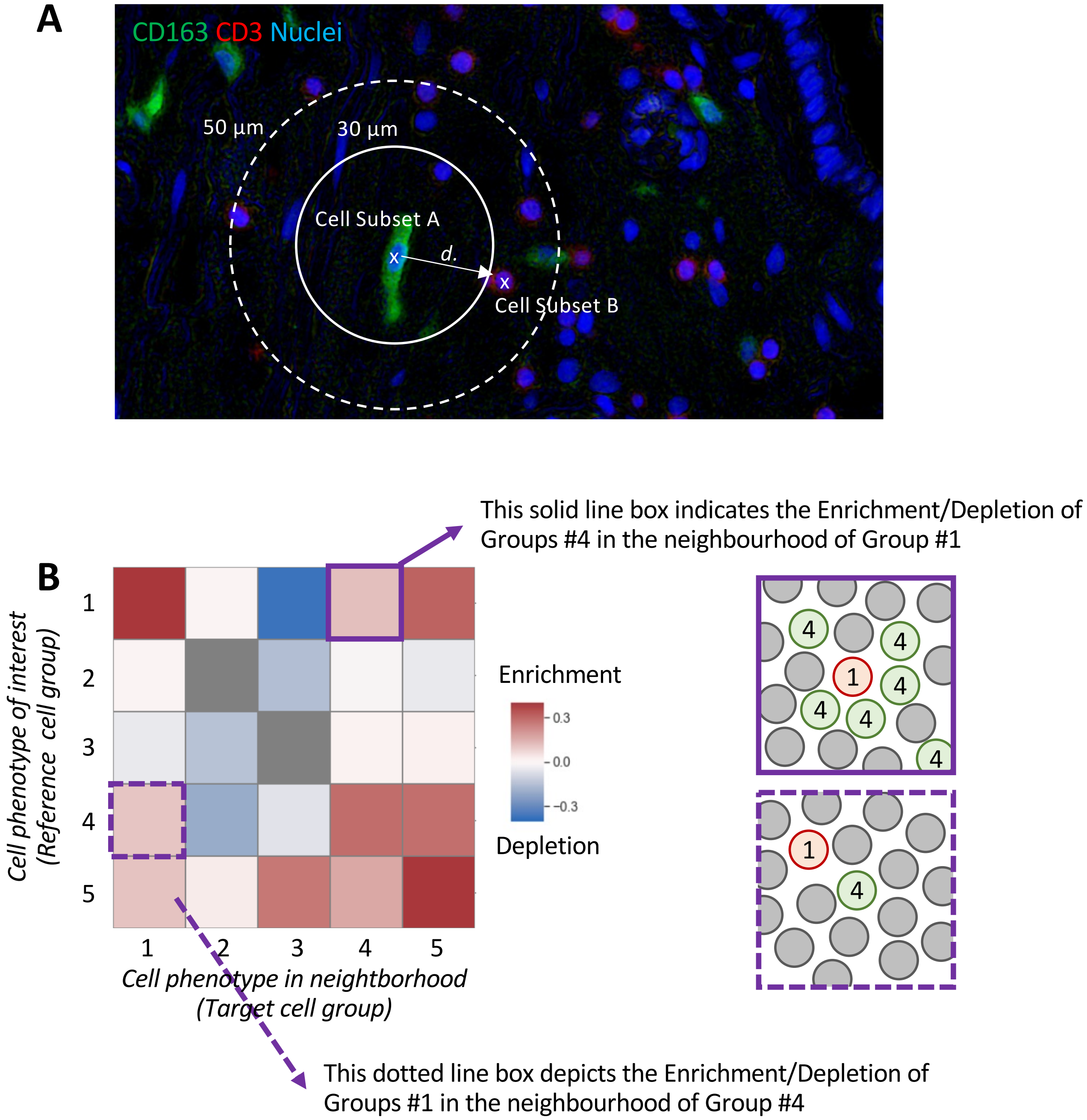

Figure S9. (A) Representation image (simulated IF) showing radius of 30 µm and 50 µm. *d.* represents one shortest distance from one cell of Subset A to one cell of Subset B; the average shortest distance from Subset A to B was calculated as the average value of *d.* Note the average shortest distance from A to B is a different value than from B to A. (B) Example of neighbourhood enrichment analysis. The heatmap represents the enrichment or depletion of a given target cell group in the neighbourhood of a given reference cell group (cell phenotype of interest) comparing with a random background; Likelihood of enrichment or depletion was represented by red or blue, respectively; grey indicates the likelihood is not significant.

Supplementary figures

Figure S10

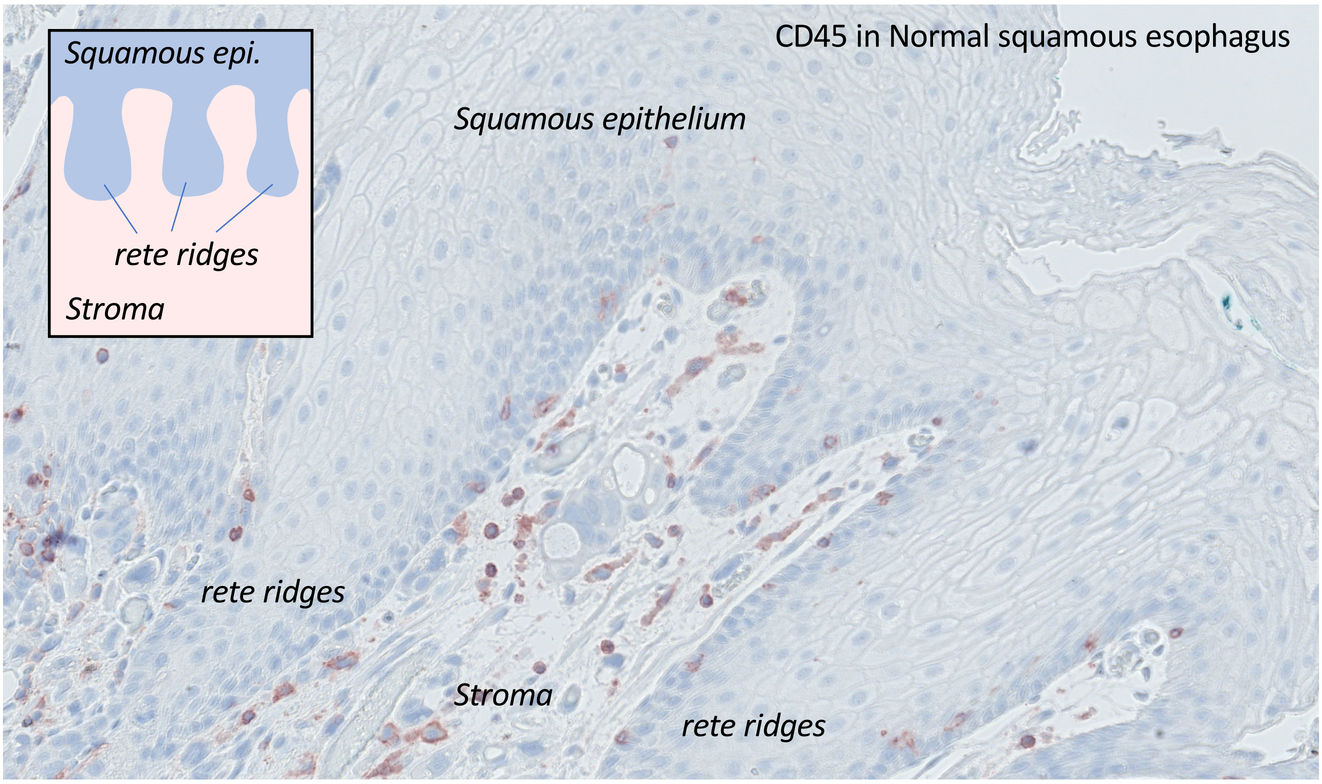

Figure S10. Schematics and simulated CD45 IHC of normal squamous esophagus. Note the undulating pattern of rete ridges, and the enriched $CD45^{+}$ cells.
